# Controlled *Mycobacterium tuberculosis* infection in mice under treatment with anti-IL-17A or IL-17F antibodies, in contrast to TNFα neutralization

**DOI:** 10.1038/srep36923

**Published:** 2016-11-17

**Authors:** Noria Segueni, Elaine Tritto, Marie-Laure Bourigault, Stéphanie Rose, François Erard, Marc Le Bert, Muazzam Jacobs, Franco Di Padova, Daniel P. Stiehl, Pierre Moulin, Dominique Brees, Salah-Dine Chibout, Bernhard Ryffel, Michael Kammüller, Valerie F. Quesniaux

**Affiliations:** 1 CNRS, UMR7355, Orleans, France; 2 University of Orleans, INEM, Experimental and Molecular Immunology and Neurogenetics, Orleans, France; 3 Novartis Institutes for Biomedical Research, CH-4002, Basel, Switzerland; 4 Division of Immunology, Institute of Infectious Disease and Molecular Medicine, Health Sciences Faculty, University of Cape Town, South Africa; 5 National Health Laboratory Service, Cape Town, South Africa

## Abstract

Antibodies targeting IL-17A or its receptor IL-17RA show unprecedented efficacy in the treatment of autoimmune diseases such as psoriasis. These therapies, by neutralizing critical mediators of immunity, may increase susceptibility to infections. Here, we compared the effect of antibodies neutralizing IL-17A, IL-17F or TNFα on murine host responses to *Mycobacterium tuberculosis* infection by evaluating lung transcriptomic, microbiological and histological analyses. Coinciding with a significant increase of mycobacterial burden and pathological changes following TNFα blockade, gene array analyses of infected lungs revealed major changes of inflammatory and immune gene expression signatures 4 weeks post-infection. Specifically, gene expression associated with host-pathogen interactions, macrophage recruitment, activation and polarization, host-antimycobacterial activities, immunomodulatory responses, as well as extracellular matrix metallopeptidases, were markedly modulated by TNFα blockade. IL-17A or IL-17F neutralization elicited only mild changes of few genes without impaired host resistance four weeks after *M. tuberculosis* infection. Further, the absence of both IL-17RA and IL-22 pathways in genetically deficient mice did not profoundly compromise host control of *M. tuberculosis* over a 6-months period, ruling out potential compensation between these two pathways, while TNFα-deficient mice succumbed rapidly. These data provide experimental confirmation of the low clinical risk of mycobacterial infection under anti-IL-17A therapy, in contrast to anti-TNFα treatment.

Antibodies targeting IL-17A or IL-17RA show unprecedented efficacy in the treatment of several autoimmune diseases, and have been approved for psoriasis, psoriatic arthritis, and ankylosing spondylitis^1^,^2^,^3^. However, these therapies, by neutralizing critical mediators of innate and adaptive immunity, carry a risk of an increased susceptibility to specific infections, such as mucocutaneous candidiasis^4^,^5^. The importance of IL-17 cytokine family members in host resistance to intracellular *Mycobacterium tuberculosis* infections is less clear, in comparison with established anti-tumor necrosis factor (TNFα) antibody treatments, which have been associated with increased incidence of acute tuberculosis and reactivation of latent tuberculosis infection^6^,^7^.

The role of Th17 cells and IL-17A in *M. tuberculosis* infection has been addressed in mice^8^,^9^,^10^,^11^,^12^,^13^ and humans^14^,^15^,^16^. Both protective and pathologic roles have been described for Th17 cells and IL-17A in mycobacterial infection^13^. IL-17A is induced in early phases of *M. tuberculosis* infection^14^ and contributes to the recruitment of neutrophils^17^,^18^. Human and animal vaccination studies with *M. bovis* bacille Calmette-Guérin (BCG) show increased IL-17A responses^18^,^19^,^20^, but specific T cell frequency and cytokine expression profile did not correlate with protection against tuberculosis after BCG vaccination^21^. The IL-17 pathway seems to be dispensable for low dose *M. tuberculosis* host resistance^12^,^13^, while control of a higher inoculum of *M. tuberculosis* H37Rv^10^,^11^ or of the hypervirulent *M. tuberculosis* strain HN878 appears to induce an IL-17A-dependent inflammation, as recently described^12^. The IL-17RA pathway is critical in CXCL1 and CXCL5-mediated early neutrophil recruitment following *M. tuberculosis* H37Rv infection^17^.

The physiological importance of the Th17/IL-17 pathway in immune surveillance of (mostly extracellular) pathogens at mucocutaneous barrier tissues^22^ triggered an inquiry into a potential role of IL-17A in host resistance to *M. tuberculosis*. CD4^+^ T cells, IFNγ, TNFα, IL-12p40, together with the IL-1/IL-1R1 pathway, nitric oxide, reactive oxygen and reactive nitrogen intermediates, are essential to control intracellular *M. tuberculosis* infection^23^,^24^. The importance of TNFα in *M. tuberculosis*-triggered immune responses, identified in mouse studies early on in the field of experimental tuberculosis research^25^, has also been documented by numerous clinical studies^6^,^7^. While TNFα from myeloid cells^26^ and TNFR1 on innate macrophage and neutrophil myeloid cells mediates early host resistance^27^, T cell-derived TNFα is essential to sustain protection during chronic mycobacterial infection in mice^26^. Indeed, mice deficient for TNFα or TNFR1 on myeloid cells exhibited 2 log_10_ increased pulmonary bacterial load 4 weeks post-*M. tuberculosis* infection, as compared with wild-type mice^26^,^27^, while mice deficient for TNFα of T cell origin succumbed by day 150 after *M. tuberculosis* infection (200–500 CFU/lung) with severe lung histopathology, necrosis and occluded alveolar space, and significantly increased, 10-fold higher pulmonary bacterial load, as compared with wild-type mice^26^.

Th17 cells were originally shown to produce IL-17A, IL-17F, but also IL-21 and IL-22^28^,^29^. IL-17 and IL-22 play an important role coordinating pulmonary immune defense, with IL-17 and IL-22 primarily acting on the lung epithelium, inducing antimicrobial proteins and neutrophil chemoattractants^29^,^30^. Although IL-17A and IL-17F share many functions^29^, different activities and cooperation between IL-17A and IL-17F have been reported^17^,^31^. Recently, differential roles of IL-17A and IL-17F have been documented in a murine model of acute oral mucosal candidiasis^32^, but the role of IL-17F in murine *M. tuberculosis* infection has not been addressed yet. IL-17 and IL-22 are produced by cells from the innate (LTi and ILC3) and adaptive immune system (Th17 and Th22) in response to RORγT transcription factor binding to their promoter region^33^. Distinct IL-17- and IL-22-producing CD4^+^ T cell subsets appear to contribute to human^34^ and bovine^20^ anti-mycobacterial immune responses, and local concentrations of IL-22 exceed IL-17 in *M. tuberculosis* infected patients, supporting a role for IL-22 in tuberculosis-induced pathology or repair^35^. In mice, IL-22 is mainly produced by IFNγ-secreting cells, however, is dispensable for host protection against *M. tuberculosis* infection as seen in IL-22^−/−^ mice^36^ and anti-IL-22-antibody-treated mice^37^. However, a possible compensation of IL-22 by IL-17A or IL-17F has been proposed in host resistance to *M. tuberculosis* infection^36^. A defective control of *M. tuberculosis* infection has recently been reported in humans and mice deficient for RORγt with abolished IL-17A, IL-17F and IL-22, and a selective defect in IFNγ production^38^.

Reports that IL-17A-producing γδT cells and CD4^+^ T cells play a potential role during different phases of *M. tuberculosis* infection^8^,^9^,^10^,^11^,^12^,^13^, emphasize the need to further explore the role of this cytokine (family)^13^, in comparison with TNFα. The role of TNFα, IL-17A and IL-22 in murine host immune responses to *M. tuberculosis* infection have been studied separately. The present investigation compares the effect of anti-IL-17A or TNFα neutralizing antibodies side-by-side, including TNFα-deficient mice as susceptible controls, and in addition the role of IL-17F, in a commonly used *M. tuberculosis* H37Rv strain infection model. We correlated comprehensive gene expression analysis with pathological assessments, and also investigated the effect of IL-17A neutralization in a reactivation model, using TNFα-deficient mice as susceptible controls. Any potential compensation between IL-17A, IL-17F and IL-22 pathways during early and late phases of *M. tuberculosis* infection was studied in IL-17RA-, IL-22- and double IL-17RA-IL-22-deficient mice. Our studies further support the concept that neither IL-17 nor IL-22 pathways are central for controlling *M. tuberculosis* infection, unlike TNFα.

## Results

### Controlled *M. tuberculosis* infection under treatment with anti-IL-17A or anti-IL-17F antibodies, in contrast to TNFα neutralization

**Figure 1:**
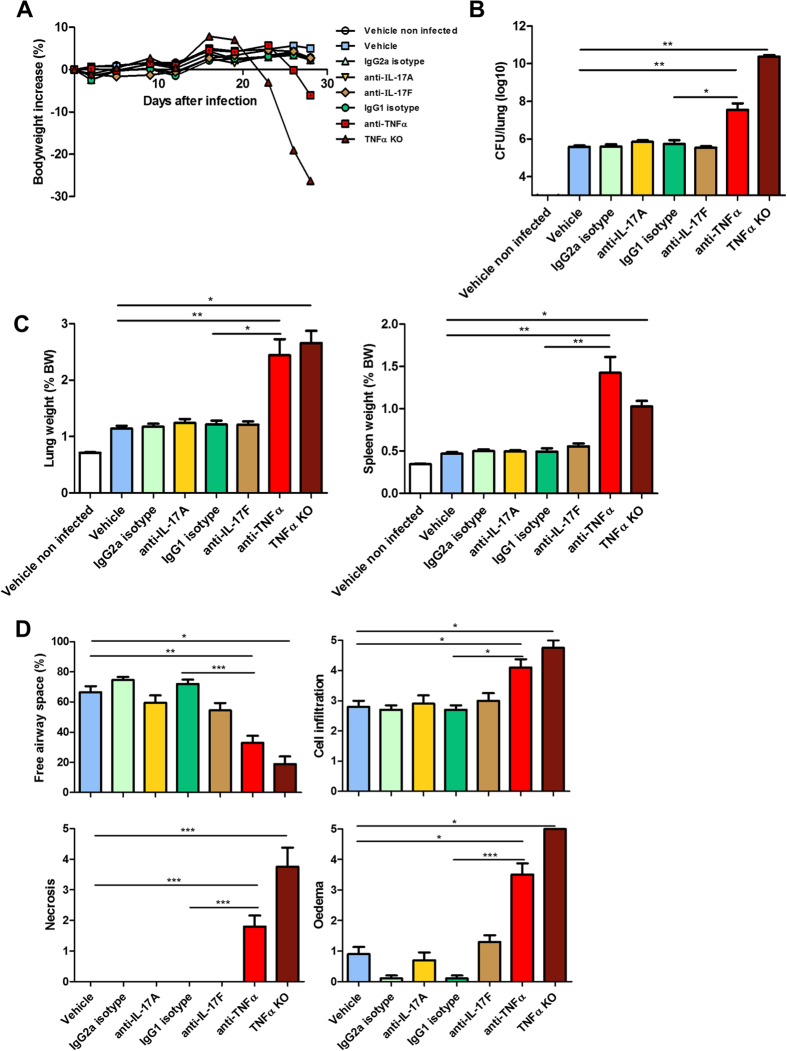
Neutralizing IL-17A or IL-17F does not compromise host response to early phase of *M. tuberculosis* infection, in contrast to TNFα neutralization. *M. tuberculosis* infected C57BL/6 mice (H37Rv, 1000 CFU i.n.) were treated once per week with anti-mouse IL-17A or IL-17F antibodies (20 mg/kg i.p.), anti-mouse TNFα antibody (10 mg/kg), respective isotype control antibodies, or vehicle, starting 1 day before the infection, and monitored for body weight (**A**). Pulmonary bacterial load (**B**), lung and spleen relative weights (**C**) were measured on day 28 post-infection. (**D**) Lung pathology was assessed on day 28. Histopathological scores of free alveolar space, cell infiltration, necrosis and oedema were determined by two independent observers. Results are expressed as mean +/− SEM of n = 10 treated mice per group and n = 4 TNF KO mice, from one experiment representative of two independent experiments. *p < 0.05, **p < 0.01, ***p < 0.001 as compared with vehicle or isotype controls.

To assess whether blocking the IL-17 cytokine family axis may affect host susceptibility to *M. tuberculosis* H37Rv strain infection, we investigated the effect of neutralizing IL-17A or IL-17F during acute infection, in comparison to TNFα neutralization (Figure S1A). We used anti-IL-17A or IL-17F antibody (500 μg/mouse, 20 mg/kg) or anti-TNFα antibody (250 μg/mouse, 10 mg/kg) or the respective isotype control IgG antibodies, administered weekly, starting 1 day before infection. The anti-mIL-17A antibody (rat IgG2a; MAB421 R&D; clone 50104) used here, was previously shown to be active during several weeks in different mouse models^39^,^40^; the anti–IL-17F antibody (rat IgG1; 16–7473 e-bioscience; clone RN17), effective in other mouse infection models^32^ could be directly compared with an anti-mTNFα antibody of the same isotype (rat IgG1; MAB4101 R&D; clone MP6-XT22) utilized earlier in murine *M. tuberculosis* infection experiments^41^,^42^. The specific activities of these anti-IL-17A and IL-17F neutralizing antibodies used here were shown in a murine model of acute oral mucosal candidiasis^32^, and allergic asthma (Ryffel B *et al.*, in preparation). In the experiments depicted in Fig. 1, anti-IL-17A antibody plasma levels were clearly detectable in most treated mice after four weeks, but not in isotype control-treated animals (Supplementary Fig. S2). However, administration of anti-mouse IL-17A and IL-17F neutralizing antibodies or their respective IgG2a and IgG1 isotype controls, had no effect on body weight development (Fig. 1A) and pulmonary bacterial loads (Fig. 1B) by day 28, in comparison to infected vehicle–treated control mice. Treatment with the anti-TNFα antibody led to a decrease of body weight and a significant increase in lung bacterial load during the fourth week of treatment, in comparison to anti-IgG1 isotype or vehicle control mice, reminiscent of the highly susceptible TNFα-deficient mice (Fig. 1B). The bodyweight decrease is a correlative non-invasive parameter reflecting the ‘clinical’ status of the immunosuppressed animals with increased bacterial burden, akin cachexia in immunosuppressed human subjects suffering from tuberculosis. Therefore, antibody neutralization of IL-17A or IL-17F had no visible effect on host resistance to early stages of *M. tuberculosis* infection, in conditions where anti-TNFα antibody treatment compromised the control of pulmonary bacterial burden.

### Neutralizing IL-17A or IL-17F has no overt effects on lung inflammation in response to *M. tuberculosis* infection, whereas blocking TNFα causes necrotic pneumonia

Relative lung and spleen weights, a first estimate of inflammation, were not increased in the groups treated with anti-mouse IL-17A or IL-17F neutralizing antibodies as compared with the isotype or vehicle-treated controls (Fig. 1C), while it was significantly increased after anti-mouse TNFα-treatment or in TNFα-deficient mice. Macroscopically, the pleural surface of infected mice had a nodular appearance, which was most prominent in the TNFα-deficient mice and anti-TNFα-antibody-treated group (Supplementary Fig. S3). These changes were associated with a worsening of the microscopic observations in the lungs of the anti-TNFα-antibody-treated animals, and showed distinct reduction of free alveolar space, concurrent with high inflammatory cell infiltration in the lungs, oedema and necrosis at week 4 (Fig. 1D). The inflammatory parameters in mice treated with IL-17A or IL-17F neutralizing antibodies were comparable to those of vehicle or isotype controls, namely an inflammatory response dominated by infiltration of macrophages, lymphocytes, as well as some neutrophils, granuloma formation occupying about 30% of the airway space, with no signs of necrosis and little oedema (Fig. 1D, Supplementary Fig. S3). Thus, IL-17A or IL-17F neutralization had no overt effect on lung inflammation, while anti-TNFα antibody treatment exacerbated inflammation in early stages of *M. tuberculosis* infection.

### TNFα neutralization exacerbates gene expression of inflammatory markers in *M. tuberculosis* infection, in contrast to anti-IL-17A or anti-IL-17F treatment

**Figure 2:**
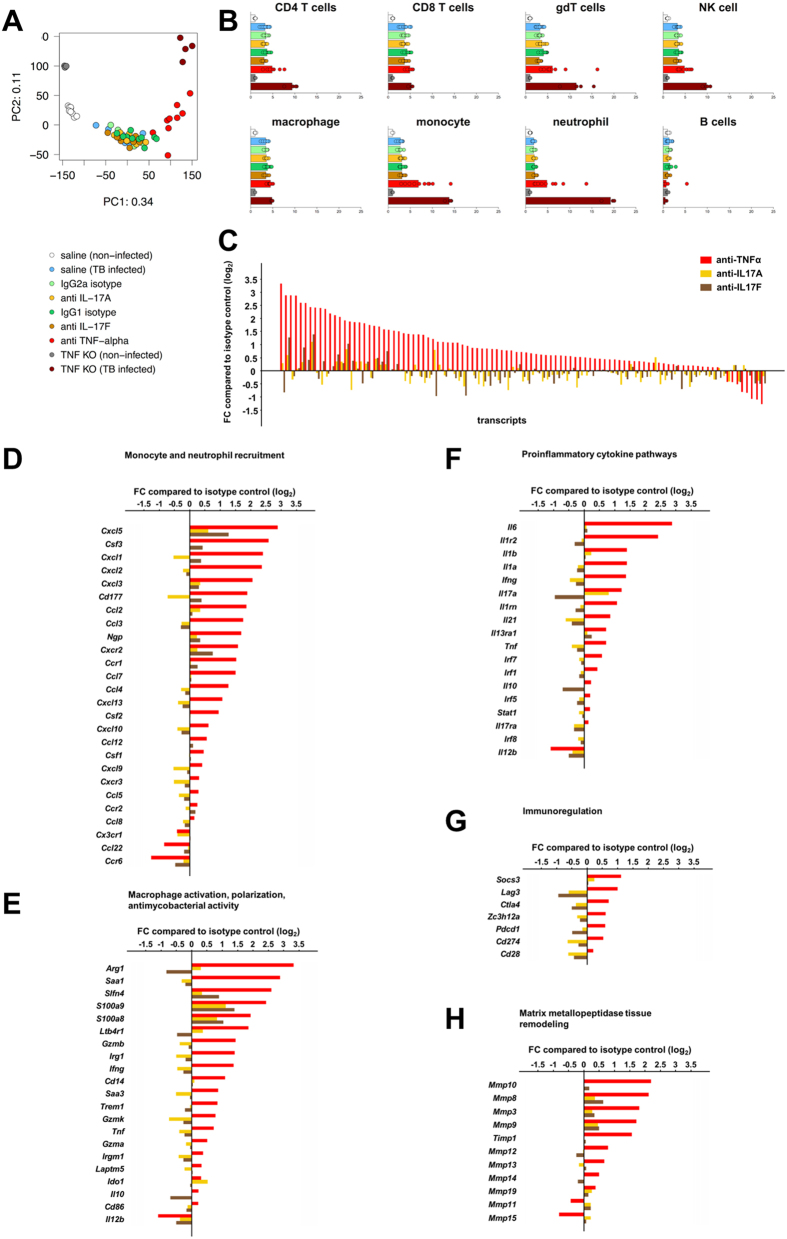
TNFα neutralization exacerbates gene expression of inflammatory markers in early phase of *M. tuberculosis* infection, in contrast to anti-IL-17A or anti-IL-17F treatment. Transcriptional profiling of lungs from *M. tuberculosis* infected C57BL/6 mice (H37Rv, 1000 CFU i.n.) treated once per week (starting day-1) with anti-mouse IL-17A or IL-17F antibodies (20 mg/kg i.p.), anti-mouse TNFα antibody (10 mg/kg), respective isotype control antibodies, from non-infected C57BL/6 mice treated with vehicle, or from infected and non-infected TNFα-deficient mice, was performed on day 28 to assess molecular changes. (**A**) Principal component analysis plot of the gene expression data showing the first two principal components. Each dot represents an individual animal. (**B**) Bar plot graphs showing the geometric mean for the fold changes of all the genes in the manually-curated gene lists for CD4 T cells, CD8 T cells, γδT cells, NK cells, macrophages, monocytes, neutrophils and B cells. The fold change for *M. tuberculosis* infected vehicle, IgG2a isotype, anti-IL–17A, IgG1 isotype, anti-IL–17F and anti-TNFα treated groups was calculated relative to non-infected vehicle-treated animals, while the fold change for infected TNFα-deficient animals was calculated relative to non-infected TNFα-deficient animals. (**C**–**H**) Waterfall plots showing selected gene transcripts indicative of host-pathogen interactions, monocyte, macrophage and neutrophil recruitment, proinflammatory cytokine and chemokine pathways, TNFα pathway, matrix metallopeptidase tissue remodeling, macrophage activation, polarization and antimycobacterial activity, and immunoregulatory responses, in lung tissue of *Mycobacterium tuberculosis*-infected mice at day 28 following anti-TNFα, anti-IL-17A or anti-IL-17F treatment. A comprehensive overview of changes is provided in (**C**), while (**D**–**H**) represent functionally annotated gene lists. All results are expressed as log_2_-transformed fold changes (FC) in mRNA expression of anti-TNFα, anti-IL-17A or anti-IL17F treated infected mice over the respective IgG2a or IgG1 isotype controls. Transcripts are ranked by magnitude of FC in anti-TNFα treated animals. Boxplots for selected genes showing the MAS5 normalized expression level for each individual animal, the lower and upper quartiles and the median for the group, are depicted in Supplementary Fig. S4A–D.

A whole transcriptome microarray analysis of mouse lung tissues was performed on day 28 after *M. tuberculosis* infection, and we investigated the effect of anti-IL-17A and anti-IL-17F treatments in comparison with anti-TNFα treatment in wild-type mice and with TNFα-deficient mice (Fig. 2A–H, Supplementary Tables S1–S7, Supplementary Fig. S4A–D).

#### Controls – infected

Wild type mice infected with *M. tuberculosis* showed many transcriptional changes in lung tissue, with 1306 upregulated and 451 downregulated genes (p-val ≤ 0.05 and fold change ≥|2|). Principal component analysis (PCA) clearly differentiated naïve from infected mice (Fig. 2A). Figure 2B shows changes of major cell populations of the innate and adaptive immune system based on gene signatures (see Supplementary Tables S1–8), and highlights that many lymphoid populations are activated and increased in response to *M. tuberculosis* infection when compared to uninfected controls at day 28 after infection. Specifically, transcriptional signatures corresponding to CD4^+^ T cells (Supplementary Table S1), CD8^+^ T cells (Supplementary Table S2), γδ T cells (Supplementary Table S3), NK cells (Supplementary Table S4), macrophages (Supplementary Table S5), monocytes (Supplementary Table S6), and neutrophils (Supplementary Table S7) were increased after infection. Changes at day 28 were associated (but not limited) to the modulation of genes associated with host-pathogen interactions, macrophage activation, host-antimycobacterial activities, immunomodulatory responses, as well as extracellular matrix metallopeptidases (Supplementary Fig. S4A–D). Gene modulation of selected cytokines and chemokines showed upregulated transcripts of *Cxcl1*, *Cxcl2*, *Cxcl5*, *Cxcl10*, *Ccl2*, *Tnf*, *Fasl*, *Ifng*, *Il1a*, *Il1b*, *Ilrn*, *Il1r2*, *Il6*, *Il10*, *Il12b and Il13ra1* genes in response to *M. tuberculosis* infection at day 28 (Supplementary Fig. S4A). Only minor changes were observed for expression of *Il17*, *Il17ra*, *Il22* and *Il23a* on day 28 of infection (Supplementary Fig. S4A). Gene profiles described here, which reflect the complex dynamic interplay between host and pathogen at day 28, match kinetic transcriptome profiles reported previously between day 12 and 100 in comparable mouse *M. tuberculosis* infection models^43^,^44^.

#### Anti-TNFα antibody treatment

We used anti-TNFα antibody treatment and TNFα-deficient mice as reference, and show that gene expression patterns associated with host-pathogen interactions, monocyte and neutrophil recruitment, proinflammatory cytokine pathways, macrophage activation, polarization and antimycobacterial activity, immunoregulation and tissue remodeling were clearly modulated as expected in early *M. tuberculosis* infection on day 28 (Fig. 2C–H; Supplementary Fig. S4A–D). Whereas most aforementioned cytokine and chemokine genes (*Cxcl1*, *Ccxl2*, *Cxcl5*, *Cxcl10*, *Ccl2*, *Tnf*, *Fasl*, *Ifng*, *Il1a*, *Il1b*, *Ilrn*, *Il1r2*, *Il6*, *Il10*, *Il13ra1*, and in addition *Il17* and *Il17ra*) were even further upregulated following anti-TNFα antibody treatment, some genes (e.g. *Il12b*) were actually downregulated compared to isotype controls (Fig. 2D,F; Supplementary Fig. S4A), reflecting the dysregulated host response associated with uncontrolled infection induced by TNFα blockade (Fig. 1). Some upregulated genes (e.g. *Cxcr2*, *Cxcl1*, *Cxcl3*, *Cxcl2*, *Cxcl5*, *Csf3*, *Npg* and *CD177*) clearly point to the presence of neutrophils (Fig. 2D; Supplementary Fig. S4A), whereas others indicate the presence of monocytes and macrophages (e.g. *Ccl2*, *Ccl3*, *Ccl5*)^45^. Gene expression patterns on day 28 provide only a snapshot of a dynamic process^43^,^44^, and revealed a mixed picture of macrophage pro-inflammatory M1 and a prominent anti-inflammatory M2 polarization under TNFα blockade, as exemplified by concomitant gene expression of *Nos2*, *Ifng*, *Il1*, and *Arg1*, *Il10* and low *Il12b*, respectively (Fig. 2E,F; Supplementary Fig. S4A,B)^46^,^47^. Coexpression of *Nos2*, *Arg1*, *S100a8* and *S100a9* (Fig. 2E; Supplementary Fig. S4B) may also point to the presence of myeloid-derived suppressor cells (MDSC)^48^,^49^,^50^,^51^. Furthermore, a mixed pattern of pro- and anti-apoptotic gene responses was found in anti-TNFα antibody-treated mice, including increased *Tnf*, *Fasl*, *Ripk1*, *Ripk3* gene expression (Supplementary Fig. S4A,C). Other modulated gene expression patterns have been noted (e.g. increased expression of *Irg1*^52^, *Ltb4r*^47^, *Ctla4*, *Pdcd1* (PD-1), *Cd274* (PD-L1), *Lag3*, *Socs3*, *Zc3h12a* (MCPIP)^53^; decreased expression of *Pparg*) (Fig. 2E,G; Supplementary Fig. S4B), which have been associated with macrophage immunoregulatory pathway alterations in *M. tuberculosis* infection^47^,^54^. Activation of proteolytic matrix metallopeptidases (e.g. *Mmp3*, *Mmp8*, *Mmp9*, *Mmp10*, *Mmp12*, *Mmp13*, *Mmp14*)^45^,^54^,^55^, induced by anti-TNFα antibody treatment of *M. tuberculosis-*infected mice (Fig. 2H; Supplementary Fig. S4D), are essential in destruction of lung extracellular matrix, and further contribute to necrosis and increased mycobacterial burden (Fig. 1). Overall, gene expression changes were comparable between anti-TNFα-antibody-treated and TNFα-deficient mice, or even more pronounced in the latter group.

#### Anti-IL-17A or IL-17F antibody treatment

Importantly, in direct comparison to TNFα neutralization, anti-IL-17A or IL-17F antibody treatment elicited only mild gene expression changes four weeks after *M. tuberculosis* infection (Fig. 2A–C; Supplementary Fig. S4A–D), not correlated to increased mycobacterial burden or pathological alterations (Fig. 1; Supplementary Fig. S3). Although gene expression patterns following anti-IL-17A or IL-17F antibody treatment essentially aggregated with isotype controls, and clearly segregated from anti-TNFα-antibody-treated and TNFα-deficient mice, as indicated by PCA mapping (Fig. 2A), we found a few changes upon closer examination (Fig. 2C–H). CD4 and CD8 representative signatures were slightly less induced by IL-17A or IL-17F neutralizing antibody compared to their respective isotype controls (Fig. 2B). Anti-IL-17A treatment showed increased *Il17* and lower *Il17ra* gene expression, while anti-IL-17F treatment led to reduced *Il17* expression (Fig. 2F). *Cd28*, *Ctla4*, *Cd274* (PD-L1) and *Zc3h12a* (MCPIP) showed reduced expression in both anti-IL-17A and IL-17F treatments in lung tissue at day 28 (Fig. 2G; Supplementary Fig. S4B), in contrast to TNFα blockade. Apart from enhanced *S100a8* and *S100a9* expression (Fig. 2E), anti-IL-17A and IL-17F treatments had neither marked effects on macrophage polarization, pro- and anti-apoptotic gene responses, nor on a range of other genes involved in host resistance (Fig. 2C–H; Supplementary Fig. S4A–D).

Overall, lung transcriptomic profiles in anti-TNFα-antibody-treated mice clearly reflected dysregulated immunoregulatory pathways in response to *M. tuberculosis* infection, whereas anti-IL-17A or anti-IL-17F treatment showed only few subtle effects, without impaired host resistance to mycobacterial infection.

#### Neutralizing IL-17A does not compromise host response to *M. tuberculosis* infection in a murine reactivation model

**Figure 3:**
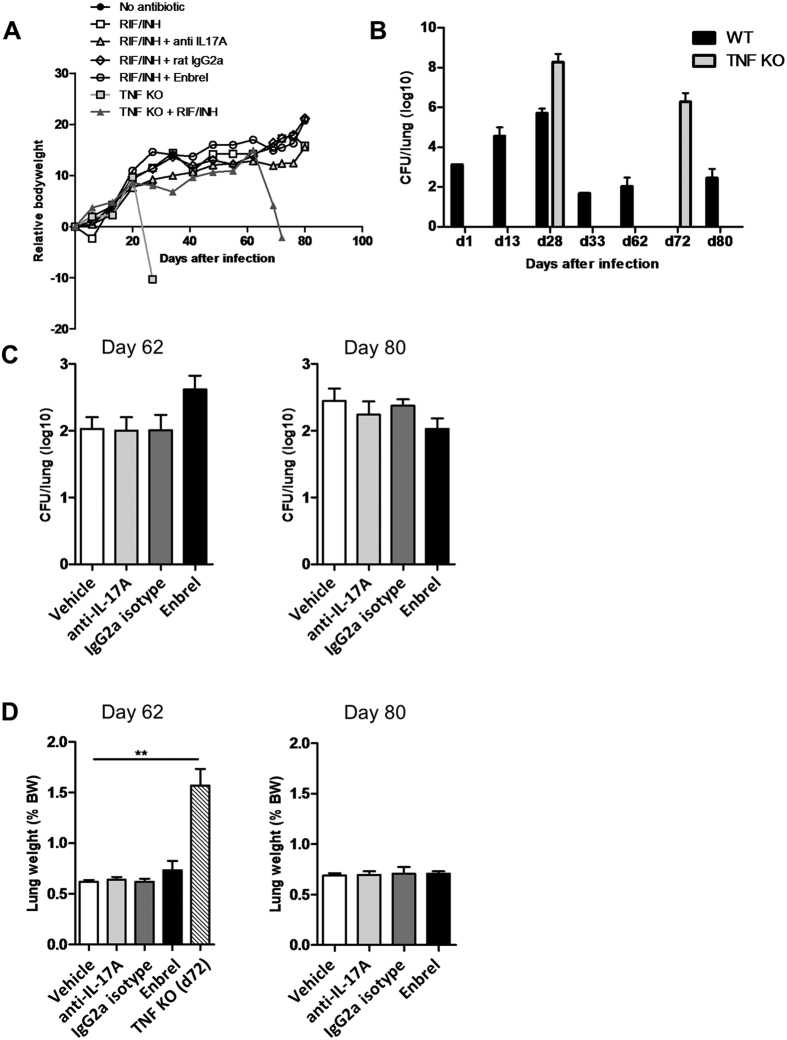
Neutralizing IL-17A does not compromise host response to *M. tuberculosis* infection in a murine reactivation model. C57BL/6 mice or TNFα-deficient mice were infected with *M. tuberculosis* infection (H37Rv, 1000 CFU i.n.); they received isoniazid (INH) and rifampicin (RIF) to control the infection, from day 14 to 35. On day 35, mice were then injected i.p. weekly with an IL-17A neutralizing antibody (IgG2a; MAB421 R&D), or the respective isotype control antibody (MAB 006 R&D; all 1 mg/mouse) or with Enbrel (30 mg/kg), starting on day 35 for 4 weeks, until day 62. Body weight (**A**) and pulmonary bacterial load (**B**) in wild-type and TNFα-deficient mice were monitored during 80 days. (**B**) Depicts several protocol controls, namely original bacterial burden on day 1, increased burden in wild-type mice on day 13 and 28 in the absence of antibiotics treatment, exacerbated bacterial load in untreated TNF KO mice on day 28 when they had to be terminated; The effectiveness of the INH+RIF regimen was verified in wild-type mice on day 33, with a slow recovering bacterial growth thereafter measured in wild-type mice on day 62 and 80, and in TNF KO on day 72 when their condition rapidly degraded and they had to be terminated. Lung bacterial load (**C**) and relative weight (**D**) in the anti IL-17A antibody or Enbrel-treated groups, with saline and isotype controls were measured on day 62 and 80 post-infection; relative lung weight of TNFα-deficient animals on day 72 included for comparison (**D**). Results are expressed as mean +/− SEM of n = 10 mice per group. **p < 0.01 as compared with vehicle controls.

Next, we asked whether neutralizing either IL-17A or TNFα would impair the control of a reactivating *M. tuberculosis* infection^56^ (Supplementary Fig. S1B). As before, immunocompromised TNFα-deficient mice served as controls for infection reactivation (Fig. 3A). The antibiotic treatment effectively reduced lung bacterial load (from 47000 to 50 CFU/lung; Fig. 3B). While *M. tuberculosis* infected TNFα-deficient mice receiving no antibiotics developed a severe disease within 3 weeks, Isoniazid (INH) and Rifampicin (RIF) treated TNFα-deficient mice survived beyond 8 weeks post infection (Fig. 3A). Four to five weeks after antibiotic treatment, TNFα-deficient mice rapidly lost weight, developed severe illness, and were terminated at 10 weeks with lung bacterial loads of 3 × 10^6^ CFU/lung (Fig. 3B), severe inflammation and necrotic pneumonia, displaying distinct morphological signs of *M. tuberculosis* infection reactivation (Fig. 3C,D, Supplementary Fig. S5). In wild-type mice, after infection and INH and RIF treatment, anti-IL-17A antibody treatment for four weeks did not induce any signs of inflammation or reactivation of infection (Fig. 3C,D), whereas TNFα-deficient mice and Enbrel (TNFα-neutralizing TNFR2 fusion molecule)-treated mice showed marked to moderately increased levels of mycobacterial burden, respectively. Thus, in a model of infection and chemotherapy adequate to observe reactivation of *M. tuberculosis* infection in the complete absence of TNFα, there was no major effect of IL-17A neutralization.

#### Contained **M. tuberculosis** infection in the absence of both IL-17RA and IL-22 pathways

**Figure 4:**
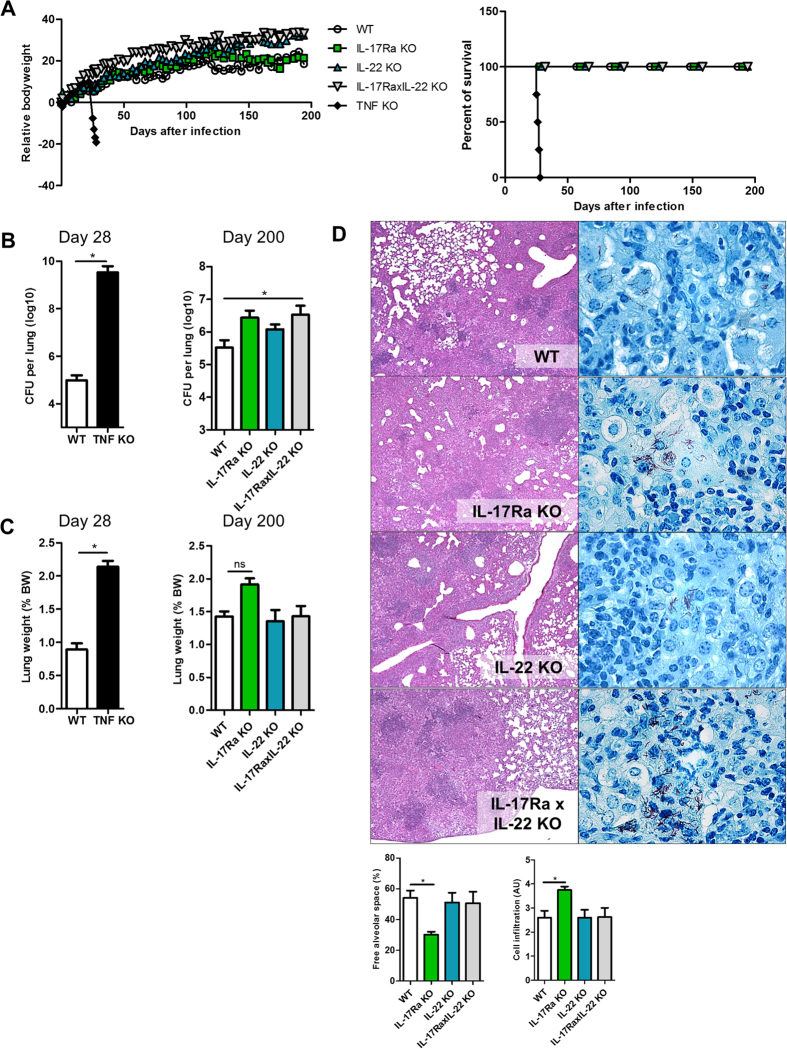
Contained chronic *M. tuberculosis* infection in the absence of IL-17RA and/or IL-22 pathways. Mice deficient for IL-17RA, IL-22, both IL-17RA and IL-22, and TNFα mice as well as wild-type mice were infected with *M. tuberculosis* (H37Rv, 1000 CFU/mouse i.n.). Body weight and survival were monitored during 200 days (**A**). Pulmonary bacterial load (**B**) and lung relative weight (**C**) were measured 28 days post-infection for sensitive TNFα-deficient mice and 200 days post-infection for the IL-17RA- and/or IL-22-deficient mice. Lung pathology was assessed at 6 months (**D**). Macroscopically large, confluent nodules were visible in all groups. Microscopic examination showed extensive inflammation with limited free alveolar space (Left, haematoxylin and eosin, magnification ×50), with the presence of acid-fast bacilli (Right, Ziehl-Nielson, magnification ×1000). Histopathological score of free alveolar space and cell infiltration per group are indicated. Results are expressed as mean +/− SEM of n = 4–7 mice per group, *p < 0.05.

**Figure 5:**
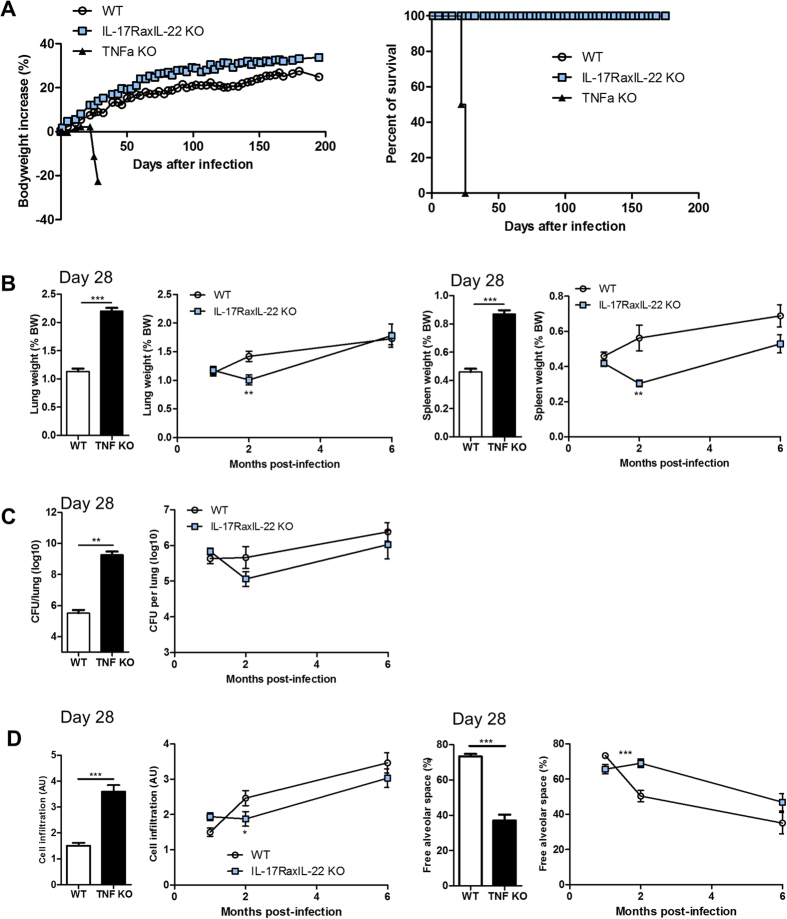
Kinetics of lung *M. tuberculosis* control and inflammation in double IL-17RA-IL-22-deficient mice. Mice deficient for both IL-17RA and IL-22, TNFα deficient mice and wild-type mice were exposed to *M. tuberculosis* as above and monitored for body weight and survival (**A**). Lung and spleen relative weights (**B**), and pulmonary bacterial load (**C**) were measured at 1 month for TNFα-deficient mice and up to 6 months post-infection for IL-17RA-IL-22-deficient and wild-type control mice. Results are expressed as mean +/− SEM (n = 13–14 from 3 experiments at 1 month, n = 7–8 from 2 experiments at 2 months and n = 11–12 from 3 experiments at 6 months). Bar graphs summarise scoring of cell infiltration in the parenchyma and free alveolar space at these time points (**D**) (n = 7–9 mice per group from 2 independent experiments).

*Il22* was not induced following *M. tuberculosis* infection at day 28 in this study (Supplementary Fig. S4A), and seems to be dispensable for host protection against *M. tuberculosis* infection as seen in IL-22^−/−^ mice^36^. Since compensation of IL-22 by IL-17A or IL-17F has been proposed in host resistance to *M. tuberculosis* infection^36^, we addressed this possibility by investigating early and late stages of *M. tuberculosis* infection in IL-17RA-, IL-22- and double IL-17RA-IL-22-deficient mice (Supplementary Fig. S1C). IL-17RA, IL-22 and double IL-17RA-IL-22-deficient mice contained chronic *M. tuberculosis* infection as they survived the six-month duration of the experiment (Fig. 4A–C), while susceptible, TNFα-deficient control mice succumbed rapidly to aerogenic *M. tuberculosis* H37Rv within 4 weeks, with very high bacterial lung burden and inflammation (Fig. 4B,C). After six months the relative lung weight of IL-17RA-deficient mice was slightly higher than that of wild type, IL-22 or double IL-17RA-IL-22-deficient mice (Fig. 4C). In line with their somewhat increased lung weight, IL-17RA^−/−^ -deficient mice exhibited reduced free airway space and enhanced inflammatory cell infiltration (Fig. 4D), although lung inflammation at six month was seen in all infected groups (Fig. 4D). The bacterial burden was slightly increased in IL-17RA- and double IL-17RA-IL-22-deficient mice (Fig. 4B), and acid-fast bacilli were visible in the lungs of these groups at this time-point (Fig. 4D, right panel). Double IL-17RA-IL-22-deficient mice and wild-type mice developed progressive inflammation in infected lungs at 1 and 2 months which was most prominent at 6 months, with no signs of necrosis and little oedema (Fig. 5; Supplementary Fig. S6A,B). The inflammation was milder in IL-17RA-IL-22-deficient than wild-type mice at 2 months, with higher free alveolar space and reduced inflammatory cell infiltration (Fig. 5D), a difference less prominent at 6 months when acid-fast bacilli were detected (Supplementary Fig. S6B).

**Figure 6:**
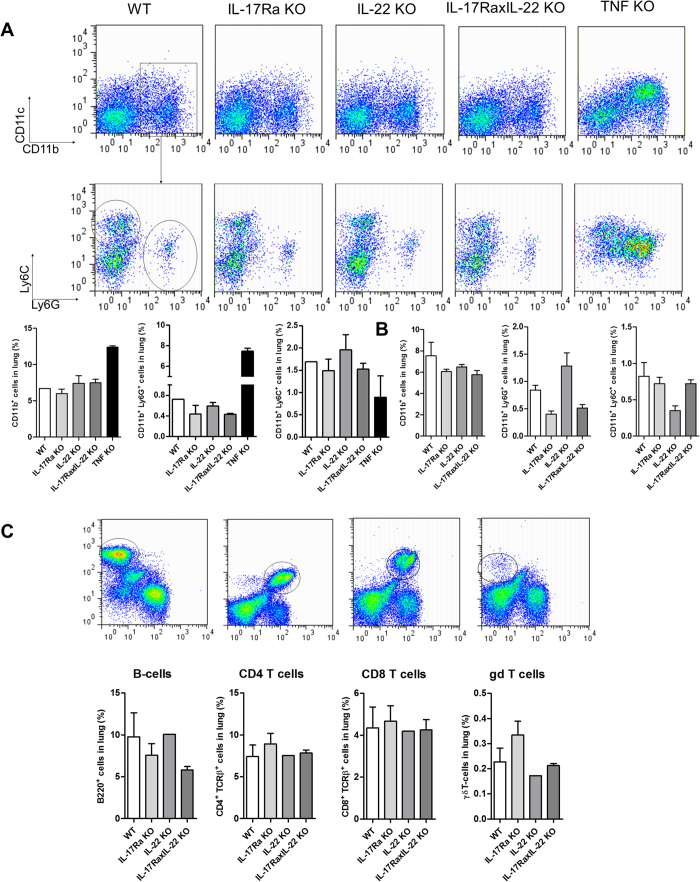
*M. tuberculosis* induced lung inflammatory cell infiltration in the absence of IL-17RA and IL-22. Mice deficient for IL-17RA, IL-22, both IL-17RA and IL-22, and TNFα mice as well as wild-type mice were infected with *M. tuberculosis* (H37Rv, 1000 CFU/mouse i.n.). Inflammatory lung infiltrating cells were analysed by flow cytometry, and bar graphs show data from 2 to 3 individual mice per group, and 2 pools of 2 TNFα-deficient mice, at 1 (**A**) or 2 months (**B**,**C**) post-infection, expressed as mean +/− SEM. Representative dot plots of CD11b^+^ and CD11c^+^ cell populations, and Ly6G^+^ and Ly6C^+^ cells gated on CD11b^+^ cells are shown (**A**). In (**C**), the gating strategy and relative populations of B220^+^, CD4^+^, CD8^+^ and γδ T lymphocyte populations are shown.

To further address the cellular inflammatory response in the absence of IL-17RA and/or IL-22 pathways, we analysed the composition of cell populations infiltrating the lung 1 and 2 months post *M. tuberculosis* infection in IL-17RA-, IL-22- and double IL-17RA-IL-22-deficient mice. Although in TNFα-deficient mice lung infiltrating cells showed an increased granulocytic CD11b^+^Ly6G^+^ cell response, the absence of IL-17RA and/or IL-22 had little influence on CD11b^+^Ly6G^+^ cells one month post-infection (Fig. 6A). CD11b^+^ CD11c^−^ Ly6C^+^ Ly6G^−^ cells, a phenotype corresponding to monocytic MDSC identified in the lung of mice susceptible to *M. tuberculosis*^49^ were slightly decreased in TNFα-deficient mice, but not affected by the absence of IL-17RA and/or IL-22 (Fig. 6A). Two months after infection the CD11b^+^Ly6G^+^ cell response was lower in the lung of IL-17RA- and IL-17RA-IL-22-deficient mice, compared to wild type mice and IL-22-deficient mice, whereas monocytic CD11b^+^Ly6C^+^ cells were decreased in IL-22-deficient mice (Fig. 6B). Similarly, decreased pulmonary granulocyte recruitment was reported in IL-17RA-deficient mice 3–5 weeks post *M. tuberculosis* infection^11^,^17^.

**Figure 7:**
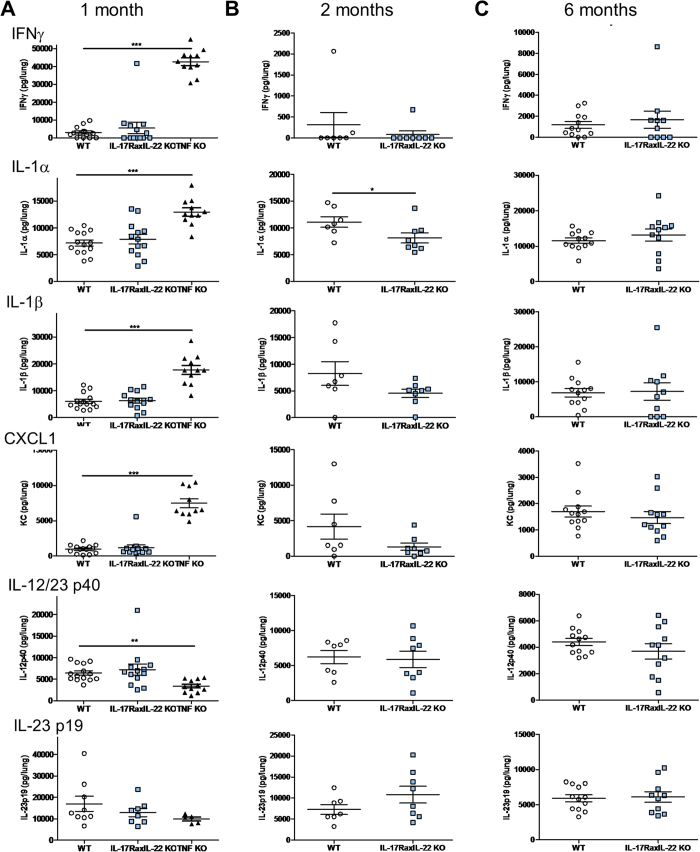
>Pulmonary cytokine levels in *M. tuberculosis* infected double IL-17RA-IL-22-deficient mice. Cytokine concentrations were determined in lung homogenates of mice deficient for IL-17RA and IL-22 and wild-type mice at 1 month (**A**), 2 months (**B**) and 6 months (**C**) post *M. tuberculosis* infection as outlined in Fig. 5. Concentrations of IFNγ, IL-1α, IL-1β, CXCL1, IL-12/IL-23 p40 and IL-23 p19 were quantified by ELISA. Results are shown for individual mice, with mean +/− SEM, and are from 2 independent experiments at 1 and 2 months and from 3 independent experiments at 6 months. *p < 0.05, **p < 0.01, ***p < 0.001.

We hypothesized that the IL-17RA and IL-22 pathways were not contributing to the early innate immune response, but rather once the adaptive response comes into play. We thus analysed the lung inflammatory response in terms of pulmonary cytokine levels in the infected lung at these time points. Pulmonary levels of IFNγ, IL-1α, IL-1β and CXCL1/KC were highly increased in TNFα-deficient mice one month after infection, when severe pathology develops, with a reduction of IL-12/23p40 and p19 (Fig. 7A), in line with transcriptome data (Fig. 2F; Supplementary Fig. S4A). No significant differences were observed in lung homogenates of double IL-17RA-IL-22-deficient mice at one month (Fig. 7A), but two months post infection there was a trend toward reduced IL-1α, IL-1β and CXCL1 lung concentrations which reached statistical significance for IL-1α (Fig. 7B), while IL-23p19 seemed to increase. This was transient and no difference in cytokine levels was detected at 6 months (Fig. 7C). Increased expression of IL-17A by CD4^+^ and γδ T-cells was reported in IL-17RA-deficient mice 5 weeks post *M. tuberculosis* infection^11^. We also addressed the influence of IL-22 on IL-17A over-expression and show that IL-17A expression was slightly increased in double IL-17RA-IL-22-deficient mice, which was associated with CD4^+^ T-cells and γδ T-cells, 2 months after *M. tuberculosis* infection (Supplementary Fig. S7).

Thus, although some subtle differences between wild type and double IL-17RA-IL-22-deficient mice were observed over a 6 months course, overall, the combined absence of the IL-17RA and IL-22 pathways did not profoundly compromise the control of chronic *M. tuberculosis* infection, and argues against major compensation between the two pathways in this model.

## Discussion

To explore the potential of anti-IL-17A antibody treatment to reactivate *M. tuberculosis* infection, we directly compared the effect of antibodies neutralizing IL-17A or IL-17F with those of a TNFα-neutralizing antibody in an acute murine *M. tuberculosis* infection model. Numerous investigative studies have documented the importance of TNFα in *M. tuberculosis*-triggered murine host responses^25^,^26^,^41^,^42^,^54^, in accordance with the clinical risk of mycobacterial infection associated with anti-TNFα therapy^6^,^7^. The importance of IL-17 cytokine family members in reactivating intracellular *M. tuberculosis* infections is less clear in comparison with the role of anti-TNFα, as both protective and pathologic roles have been described for Th17 cells and IL-17A^13^. Vaccination studies to BCG show increased IL-17A responses^19^,^20^, but specific lymphocyte and cytokine profiles did not correlate with protection against tuberculosis after BCG vaccination^21^. Reports that IL-17A-producing γδT cells and CD4^+^ T cells play a potential role during different phases of *M. tuberculosis* infection^8^,^9^,^10^,^11^,^12^,^13^,^57^,^58^, emphasize the need to further investigate the role of this cytokine (family) in comparison with TNFα.

In humans, several studies have found that *ex vivo* stimulation of whole blood or peripheral blood mononuclear cells with *M. tuberculosis* lead to IL-17 production^14^,^15^,^16^. IL-17A produced by γδT cells has been reported to play a critical role in granuloma formation in a high-dose murine *M. tuberculosis* H37Rv infection model^10^, but the IL-17 pathway seemed to be dispensable for low dose *M. tuberculosis* host resistance^12^. However, the hypervirulent *M. tuberculosis* W-Beijing strain HN878 has recently been reported to induce an IL-17A-dependent control of pulmonary bacterial burden and inflammation even at low infection doses^12^, although the overall impact on host survival was not shown. IL-17A, and to a lesser extend, IL-17F are induced in early phases of *M. tuberculosis* infection^14^,^17^, and contribute to recruitment of neutrophils^59^, which were reported to exacerbate *M. tuberculosis* infection, depending on the murine model^48^. The IL-17RA pathway was recently shown to be critical in CXCL1 and CXCL5-mediated early neutrophil recruitment after *M. tuberculosis* H37Rv infection^17^. Moreover, because IL-17- and IL-22-producing CD4^+^ T cell subsets may contribute to human anti-mycobacterial immune responses^34^,^35^,^36^, we also addressed possible compensation mechanisms between the two pathways in double IL-17RA-IL-22-deficient mice.

Here, we have utilized several independent approaches to address the significance of IL-17A and IL-17F in early and late stages of *M. tuberculosis* infection, in direct comparison with TNFα blockade. Overall, several findings from our comparative mouse studies support the concept that neither IL-17 nor IL-22 pathways are central for controlling *M. tuberculosis* infection, unlike TNFα.

First, neutralization of IL-17A or IL-17F did not compromise the host response to *M. tuberculosis* in a four week study, while TNFα neutralization resulted in a marked increase of mycobacterial burden, orders of magnitude higher than for anti-IgG1 isotype control or vehicle-treated infected C57BL/6 mice (Fig. 1B), – akin to TNFα-deficient mice (Fig. 1B), and consistent with earlier reports^25^,^26^,^41^,^42^,^54^. Furthermore, we showed that the complete absence of TNFα lead to markedly increased mycobacterial burden in a pharmacological reactivation model of *M. tuberculosis* infection (Fig. 3)^56^, whereas anti-IL-17A antibody treatment did not compromise host resistance. Also, neutralization of IL-17A with a different antibody than utilized here, did not increase susceptibility to mycobacterial BCG infection^8^. Importantly, the same neutralizing anti-mIL-17A antibody utilized here, showed effects in several mouse disease models^39^,^40^ as well on host resistance in a murine *Candida albicans* oropharyngeal infection model^32^. In this model there was no increase in fungal burden in anti-IL-17F-treated mice^32^, using the same anti-IL-17F antibody as in experiments reported here. However, the combined blockade of IL-17A and IL-17F led to increased susceptibility to oropharyngeal *Candida albicans* infection compared with either antibody alone, suggesting cooperative activity of these cytokines^32^. Hence, IL-17A and IL-17F play distinct roles in extracellular versus intracellular infections.

Secondly, while the histopathological parameters in mice treated with IL-17A or IL-17F neutralizing antibodies were comparable to those of vehicle or isotype controls, anti-TNFα-antibody-treated animals showed a distinct reduction of free alveolar space, concurrent with high inflammatory cell infiltration in the lungs, oedema and necrotic pneumonia in response to early phase *M. tuberculosis* infection at day 28 (Fig. 1D).

Third, anti-IL-17A and anti-IL-17F neutralisation had only few effects on certain host transcriptomic profiles, whereas gene expression patterns in anti-TNFα antibody-treated mice, were clearly distinct from *M. tuberculosis* infected wild-type mice at day 28 and reflect expected dysregulated immunoregulatory pathways in response to *M. tuberculosis* infection (Fig. 2A–H, Supplementary Tables S1–S7, Supplementary Fig. S4A–D). Investigations of host cytokine responses in *M. tuberculosis* infections are numerous and outcomes for specific cytokines may vary considerably depending on the species, infection model and time of sampling during an ongoing infection. Multiple host and pathogen responses, adaptations and cross-regulatory cytokine networks have been reported, which provide protection during infections, possibly favoring bacterial persistence, with minimum damage to the host^45^,^47^,^60^. The outcome of the interplay between host and pathogen is determined by immune factors acting in concert, based on gradients of cytokines and chemokines (e.g. TNFα, IL-10, IL-1, IFNγ), expression of activation and death markers on immune cells (e.g. PD-1) or abundance of enzymes (e.g. arginase-1, matrix metallopeptidases)^45^,^47^,^55^,^60^. This is reflected in the markedly altered gene expression pattern on day 28 after *M. tuberculosis* infection and anti-TNFα antibody treatment (Fig. 2 and Supplementary Fig. 4) associated with the significantly increased bacterial burdens (Fig. 1B). It clearly shows the importance of TNFα, neutrophils and macrophages in orchestrating host resistance to this infection^45^,^47^,^60^. Although TNFα from both myeloid and T lymphocytes controls early and late stages of *M. tuberculosis* infection, respectively^26^, we showed recently that the TNFR1 pathway in macrophages/myeloid cells is essential for this control^27^, whereas soluble TNFR2 down-modulates protective immune function and reduces host resistance and survival^61^. Gene expression patterns in anti-TNFα antibody-treated mice pointed towards a mixed picture of pro-inflammatory M1 (*Nos2*, *Ifng*, *Il1*) and anti-inflammatory M2 polarization (*Arg1*, *Il10* and low *Il12b*) (Fig. 2E, Supplementary Fig. 4A,B) in line with published data^46^,^47^. Genetic deficiencies in the IL-12 axis show increased susceptibility to tuberculosis^23^. Coexpression of *Nos2* and *Arg1* may also point to the presence of MDSC, which have recently been identified in the lung of mice susceptible to *M. tuberculosis*^48^,^49^,^50^. In addition, the marked increases of *S100a8* and *S100a9* (Fig. 2E; Supplementary Fig. S4B) might be associated with enhanced *Il17* expression (Fig. 2F)^51^, recruitment of neutrophils or granulocytic MDSC to *M. tuberculosis*-infected lungs, and exacerbation of pulmonary inflammation and mycobacterial burden^48^,^51^ seen following anti-TNFα antibody treatment.

Not surprisingly, negative and positive regulators of intersecting immune and metabolic responses were induced (e.g. increased expression of *Irg1*, *Ltb4r1*, *Il10*, *Il1rn*, *Ctla4*, *Pdcd1* (PD-1), *Cd274* (PD-L1), *Lag3*, *Socs3*); decreased expression of *Pparg*)^47^,^52^,^53^,^54^ (Fig. 2E–G; Supplementary Fig. S4B), in response to an increased mycobacterial burden following anti-TNFα antibody treatment. In fact, IFNγ, which was increased following anti-TNFα antibody treatment (Fig. 2F; Supplementary Fig. S4A), is a negative regulator of IL-17A during mycobacterial infections^57^. Our *in vivo* data show increased expression of *Ccl2* (MCP-1) and *Zc3h12a* (MCPIP1, Regnase-1) (Fig. 2D,G; Supplementary Fig. 4A,B) following anti-TNFα treatment, both induced by *M. tuberculosis* 38-kDa antigen *in vitro*^53^, and indicative of monocyte recruitment and macrophage M2 polarization^62^, respectively. MCPIP1, recently shown to negatively regulate IL-17-mediated signaling and inflammation^63^, may be one of several immunoregulatory factors engaged in containing infection and limiting host pathology. The minimal effect of anti-IL-17A treatment on *Ccl2* and rather a slightly decreased gene expression of *Zc3h12a* in lung tissue at day 28 (Fig. 2D,G; Supplementary Fig. S4B), suggest a dispensable role for IL-17A at this stage of the infection.

Furthermore, gene expression patterns indicate apoptotic and necrotic responses during *M. tuberculosis* infection on day 28 under TNFα blockade (Supplementary Fig. S4C). Both, deficient and excessive TNFα may lead to increased mycobacterial growth and release^64^,^65^, highlighting the key importance of TNFα in balancing cell survival, apoptosis and programmed necrosis^66^,^67^. The imbalance in these processes in anti-TNFα antibody-treated mice, may ultimately shift the protective TNFα-dependent extrinsic apoptosis pathway in lung macrophages towards the intrinsic pathway leading to *Ripk1-Ripk3*-dependent necrosis via mitochondrial reactive oxygen species^64^,^65^,^66^,^67^, and subsequent inadequate control of mycobacterial infection (Fig. 1B). Importantly, in anti-IL-17A-antibody-treated mice gene expression patterns associated with macrophage polarization and pro- and anti-apoptotic responses were not affected. In fact, a recent study showed that IL-17A actually promotes intracellular growth of *M. tuberculosis* by inhibiting apoptosis of infected macrophages^68^. Thus, while anti-TNFα antibody treatment prevents apoptosis of mycobacterially infected macrophages enabling bacterial growth, anti-IL-17A treatment may actually limit intracellular growth of *M. tuberculosis* by enhancing apoptosis of infected macrophages.

Finally, because above findings on day 28 of *M. tuberculosis* infection provide only a snapshot of a dynamic and complex process^45^,^47^, we also addressed the role of IL-17A in a chronic murine *M. tuberculosis* infection using *Il17ra* gene deficient mice. Since IL-17A may be expressed in inflammatory conditions that include other cytokines such as IL-17F and IL-22^29^, we asked whether IL-17 and IL-22 pathways are interdependent in view of their coordinating role in pulmonary immune defense^28^,^29^,^36^. IL-17RA is shared among the members of the IL-17 family in different heterodimers, IL-17A and IL-17F signalling through a complex composed of IL-17RA and IL-17RC^4^. IL-17RA and double IL-17RA-IL-22-deficient mice survived a 6-month infection, and exhibited only a slightly higher bacterial burden, in line with a previous report in IL-17RA-deficient mice^11^, however orders of magnitude lower compared to TNFα-deficient animals (Fig. 4B). TNFα neutralization was shown to reduce MDSCs’ suppressive activity and enhance their maturation into dendritic cells and macrophages^50^, and we see here a slight decrease of pulmonary monocytic CD11b^+^CD11c^−^Ly6C^+^Ly6G^−^ cells in TNFα-deficient mice, which were unaffected in the absence of IL-17RA and/or IL-22 one month post-infection. The reduced lung pathology and inflammatory responses at 2 months post-infection in double IL-17RA-IL-22-deficient mice was associated with a reduced infiltration of granulocytic CD11b^+^Ly6G^+^ cells in the lung, akin to earlier reports^49^. Decreased pulmonary neutrophil recruitment was reported 3–5 weeks post *M. tuberculosis* infection in IL-17RA-deficient mice, coincident with CD4^+^, γδ T-cells or NK recruitment to the infected lung^11^,^17^, and increased pulmonary IL-17A and IFNγ production in infected wild-type mice^17^. Moreover, we show that double IL-17RA-IL-22-deficient mice express slightly higher levels of IL-17A, mostly in γδ and CD4^+^ T cells^69^, in line with an earlier report regarding IL-17RA-deficient mice^11^ (Supplementary Fig. 7). γδ T-cells were increased two months after infection in IL-17RA-deficient mice, but not in IL-22- or double IL-17RA-IL-22-deficient mice, as compared to wild-type mice (Fig. 6C). Thus our results further link pulmonary γδ T-cells and neutrophil recruitment to the IL-17RA, but not the IL-22 pathway, in host responses to *M. tuberculosis*. Others have reported that *Il17ra*-deficient mice were not more susceptible to *M. tuberculosis* infection than controls (at a 10-fold lower infection dose than used here)^70^. Even at higher infection doses, the combined absence of the IL-17RA and IL-22 pathways did not profoundly compromise the control of chronic *M. tuberculosis* infection, which argues against major compensation between the two pathways in this model.

It was recently reported that humans and mice deficient for RORγt, having a profound reduction of IL-17A, IL-17F and IL-22 producing leukocytes, exhibit a defective control of *M. bovis* BCG and *M. tuberculosis* infection^38^. However, the susceptibility associated with the RORC mutation was attributed to a selective defect in a *M. tuberculosis*-specific IFNγ response^38^, in line with absence of mycobacterial infections in humans deficient for IL-17RA and IL-17F^4^,^5^, as well as our current demonstration that both IL-17A, IL-17F and IL-22 are largely dispensable for the control of *M. tuberculosis* infection.

In conclusion, anti-IL-17A or IL-17F antibody blockade did neither compromise early host responses to *M. tuberculosis* infection nor markedly alter gene expression, in contrast to TNFα neutralization. Our comparative mouse studies confirm that IL-17 and IL-22 pathways are not central to control early and late phases of *M. tuberculosis* H37Rv strain infection. In accordance with clinical data, these murine data provide experimental confirmation of the low clinical risk of mycobacterial infection under anti-IL-17A therapy^71^, in contrast to anti-TNFα treatment.

## Methods

### Mice

IL-17RA-72, IL-22-73 and double IL-17RA-IL-22-deficient mice obtained by intercrossing, mice deficient for TNFα exon 1 and 2^74^ backcrossed at least 10 times on C57BL/6 genetic background were bred in the UPS44 animal facility (CNRS, Orleans). For experiments, adult (8–12 week old) animals were kept in isolators in a biohazard animal unit, monitored daily for clinical status and weighed twice weekly.

### Ethics statement

All animal experiments complied with the French Government’s animal experiment regulations and were approved by the “Ethics Committee for Animal Experimentation of CNRS Campus Orleans” (CCO) under N° CLE CCO 2012–1001.

### Mycobacteria and infection

*M. tuberculosis* H37Rv (Pasteur) aliquots kept frozen at −80 °C were thawed, diluted in sterile saline containing 0.05% Tween 20, and clumping disrupted by 30 repeated aspirations through a 26-gauge needle (Omnican, Braun, Germany). Pulmonary infection with *M. tuberculosis* H37Rv was performed by delivering 1000 ± 300 CFU bacteria into the nasal cavities (20 μl each nostril) under xylazine-ketamine anaesthesia, and the inoculum size was verified 24 h after infection by determining bacterial load in the lungs of 2 wild-type control mice.

### Antibody treatment

An anti-mIL-17A antibody (rat IgG2a; MAB421 R&D; clone 50104), used previously^39^ and shown to have anti-arthritic activity during a 6–7 week treatment^40^ was injected intraperitoneally at 500 μg (20 mg/kg), starting 1 day before the infection and subsequently once per week into *M. tuberculosis* H37Rv-infected mice (Supplementary Figure 1A). Another group of mice was treated with an anti-mIL-17F (rat IgG1, 16–7473 e-bioscience; clone RN17) antibody according to above treatment schedule. An anti-mTNFα antibody (rat IgG1; MAB4101 R&D; clone MP6-XT22), which has been used previously in murine *M. tuberculosis* infection experiments^41^,^42^, was injected intraperitoneally at 250 μg (10 mg/kg). Respective isotype control rat IgG2a (MAB006: R&D; anti-KLH) and rat IgG1 (MAB005: R&D; anti-KLH) antibodies were administered according to aforementioned schedule. A parallel group of naïve, non-infected mice served as baseline control. To confirm exposure, plasma anti-IL-17A antibody levels were determined by ELISA.

### Reactivation model

In a pharmacological model of *M. tuberculosis* infection reactivation adapted from the original Cornell model^56^, mice were infected with *M. tuberculosis* H37Rv (1000 CFU i.n.) for 2 weeks, and treated with isoniazid (INH) and rifampicin (RIF) (each 0.1 g/L in drinking water) for 3 weeks to control the infection, as described^56^ (Supplementary Fig. 1B). Under this protocol, the bacterial burden is drastically reduced, and the infection may reactivate thereafter in the presence of a primed anti *M. tuberculosis* immune response. At 5 weeks, mice were injected intraperitoneally with aforementioned IL-17A neutralizing antibody, Enbrel (30 mg/kg i.p.; a TNFα-neutralizing TNF receptor (TNFR2) fusion molecule) or the respective isotype control antibody weekly for 4 weeks, and analyzed for reactivation of infection at 9 and 12 weeks (Supplementary Fig. 1B).

### Bacterial load in tissues

Bacterial loads in the lung of infected mice were evaluated at different time points after infection with *M. tuberculosis* H37Rv as described^75^. Organs were weighed and defined aliquots were homogenized in PBS in Dispomix homogenizer. Tenfold serial dilutions of organ homogenates in 0.05% Tween 20 containing 0.9% NaCl were plated in duplicates onto Middlebrook 7H11 (Difco) agar plates containing 10% OADC and incubated at 37 °C. Colonies were enumerated at 3 weeks and results are expressed as log_10_ CFU per organ.

### Histopathological analysis

For histological analysis lungs from *M. tuberculosis* infected mice were removed at different time points of infection, fixed in 4% phosphate buffered formalin and paraffin-embedded. Two to 3-μm sections were stained with haematoxylin and eosin and a modified Ziehl-Neelsen method. The latter involved staining in a prewarmed (60 °C) carbol-fuchsin solution for 10 min followed by destaining in 20% sulphuric acid and 90% ethanol before counterstaining with methylene blue. Free alveolar space, lung cellular infiltration, oedema and necrosis were quantified using a semi-quantitative score with increasing severity of changes (0–5) by two independent observers including a trained pathologist (BR).

### Preparation of lung homogenates for cytokine determination

Lungs were weighed, placed in 1 mL of PBS solution in a specific sterile plastic tube and homogenized in Dispomix homogenizer for 20 sec at 6000 rpm. Homogenates were centrifuged at 14,000 rpm, the supernatants sterilized by filtration through 0.22 μm filter (Costar-Corning, Badhoevedorp, The Netherlands) and stored at −80 °C until determination of IL-1α, IL-1β, IL-12/IL-23p40, IL-23p19, IL-17A, TNFα and IFNγ levels by ELISA (Duoset R&D Systems, Abingdon, UK).

### Microarray analysis

Total RNA was extracted by a Trizol/RNeasy extraction method with a DNase treatment step to remove DNA contamination. RNA quality was determined on a 2100 Bioanalyzer (Agilent Technologies, Palo Alto, CA, USA). Target preparation was performed with 1–4 ng RNA using NuGEN Ovation Pico WTA system (NuGEN Technologies, San Carlos, CA, USA). Single primer isothermal amplification (SPIA) cDNAs were fragmented and labeled by use of the Encore Biotin Module (both NuGEN Technologies). Biotinylated cDNA (4.55 μg) was hybridized on Mouse Genome 430 2.0 Arrays (Affymetrix, Santa Clara, CA, USA). Microarray slides were stained on Fluidics Workstation 450 and scanned on Scanner 3000 (both Affymetrix). Results were normalized by use of a MAS 5.0 algorithm (Affymetrix) using a target intensity of 150. Analysis was performed with R and Bioconductor (https://www.bioconductor.org/). Differentially expressed genes were selected by Linear Models for Microarray and RNA-seq Data (LIMMA; https://www.r-project.org/), having a fold change ≥2.0 and a P value ≤ 0.05. Data are publicly available in ArrayExpress (www.ebi.ac.uk/arrayexpress) under accession number E-MTAB-5218.

### Flow cytometry analysis of lung infiltrating inflammatory cells

Lungs were perfused with 0.02% EDTA-PBS until the tissue turned white. Lung tissue was then sliced into 1 to 2 mm^3^ pieces and incubated for 45 min at 37 °C with collagenase (150 U/ml) and DNase (50 U/ml; Sigma, St Louis, MO) in RPMI 1640 (Gibco, Paisley, Scotland, UK) containing 10 mM Hepes (Gibco) and antibiotics (Penicillin 100 U/ml-Streptomycin 100 μg/ml). Single-cell suspension was obtained by vigorous pipetting and filtering through a 100 μm and 20 μm mesh and three washes in RPMI 1640 containing 5% BSA. For immunophenotyping the cells were then stained with rat antibodies anti-mouse B220-FITC (clone RA3-6B2), CD4-V500 (clone RMA-5), CD8-PerCP-Cy5.5 or APC-Cy7 (clone 53-6-7), CD11b-PerCP-Cy5.5 (clone M1/70), Ly6C-FITC (clone AL-21), Ly6G-PE (clone 1A8), and hamster antibodies anti-mouse CD3e-PE (clone 145-2C-11) and CD11c-APC (clone HL3), all from BD Pharmingen (San Diego, CA). Hamster antibodies anti-mouse βTCR-V450 (clone H57-597) and γδTCR-APC (clone GL3) were from eBiosciences (San Diego, CA). A mouse IL-17 Secretion Assay – Detection Kit (130-094-205) from Miltenyi Biotec was used. Stained cells were washed twice, fixed with 1% paraformaldehyde (FACS Lysing solution, BD) and analyzed by flow cytometry on a CANTO II analyser (Becton Dickinson). Data were processed with FlowJo software (version 7.6.5 for Windows, FlowJo LLC, Ashland, Oregon).

### Statistical analysis

Statistical analysis was performed by ANOVA or the Student’s t test. For mortality studies, analysis was performed using the logrank test. For all tests, a p value of <0.05 was considered significant.

**Publisher’s note**: Springer Nature remains neutral with regard to jurisdictional claims in published maps and institutional affiliations.

## Additional Information

**How to cite this article**: Segueni, N. *et al.* Controlled *Mycobacterium tuberculosis* infection in mice under treatment with anti-IL-17A or IL-17F antibodies, in contrast to TNFα neutralization. *Sci. Rep.* **6**, 36923; doi: 10.1038/srep36923 (2016).

**Publisher’s note**: Springer Nature remains neutral with regard to jurisdictional claims in published maps and institutional affiliations.

## Supplementary Material

Supplementary Information
