# Supplementary material for: Controlled Mycobacterium tuberculosis infection in mice under treatment with anti-IL-17A or IL-17F antibodies, in contrast to TNFα neutralization
Source: Sci Rep. 2016 Nov 17;6:36923. doi: 10.1038/srep36923 (PMC5113257; doi:10.1038/srep36923)
Supplement: Supplementary Information [file srep36923-s1.pdf]

SUPPLEMENTARY Figures and Tables

Controlled *Mycobacterium tuberculosis* infection in mice under treatment with anti-IL-17A or IL-17F antibodies, in contrast to TNFα neutralization

Noria Segueni [1,2,°], Elaine Tritto [3,°], Marie-Laure Bourigault [1,2], Stéphanie Rose [1,2], François Erard [1,2], Marc Le Bert [1,2], Muazzam Jacobs [4,5], Franco Di Padova [3], Daniel P. Stiehl [3], Pierre Moulin [3], Dominique Brees [3], Salah-Dine Chibout [3], Bernhard Ryffel [1,2,4,*], Michael Kammüller [3,*] and Valerie F. Quesniaux [1,2,*]

[1] CNRS, UMR7355, Orleans, France

[2] University of Orleans, INEM, Experimental and Molecular Immunology and Neurogenetics, Orleans, France

[3] Novartis Institutes for Biomedical Research, CH-4002, Basel, Switzerland

[4] Division of Immunology, Institute of Infectious Disease and Molecular Medicine, Health Sciences Faculty, University of Cape Town, South Africa

[5] National Health Laboratory Service, Cape Town, South Africa

[°,*] contributed equally to this work

Corresponding authors:
Valerie Quesniaux*
INEM UMR7355, 3B rue de la Ferollerie, 45071 Orleans, France
Tel : +33 2 38 25 54 39; Fax : +33 2 38 25 79 79;
e-mail: quesniaux@cnrs-orleans.fr

Michael Kammüller*
Novartis Institutes for Biomedical Research, Klybeckstrasse 141, CH-4057, Basel, Switzerland;
e-mail: michael.kammueller@novartis.com

Supplementary Figures S1 – S7

Supplementary Tables S1 – S8

Supplementary Figure 1

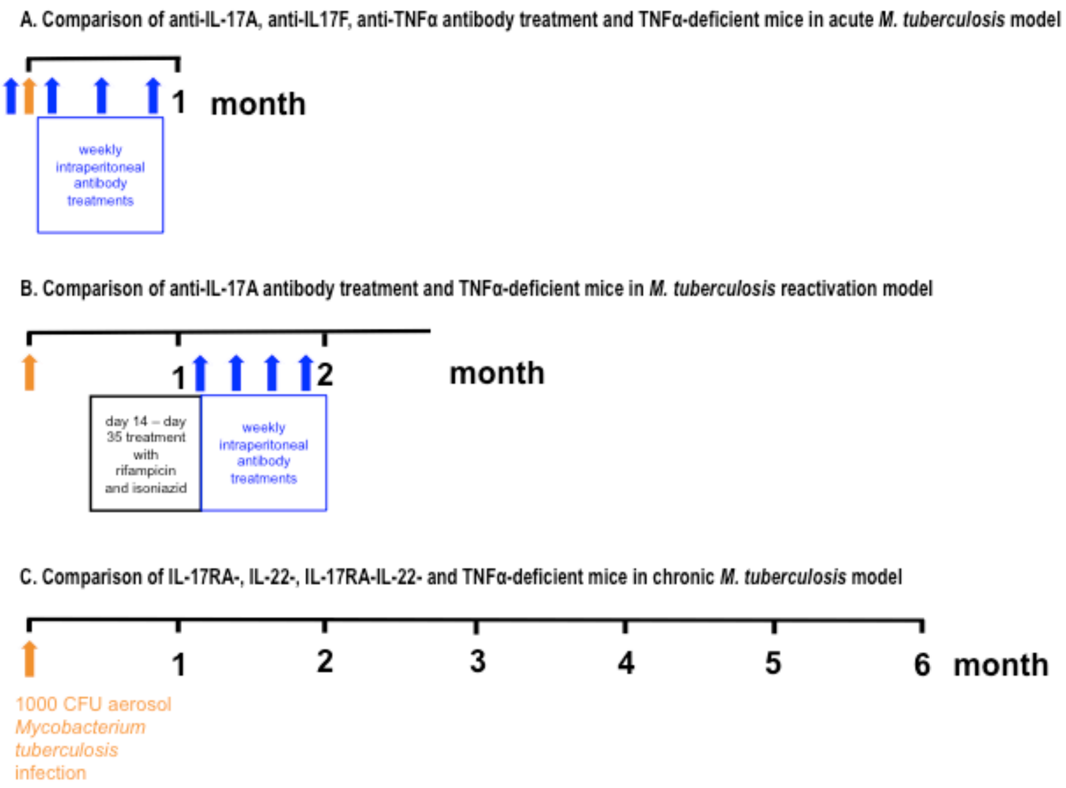

Supplementary Fig. S1. Schematic overview of utilized *M. tuberculosis* H37Rv strain infection models. (A) Comparison of anti-IL-17A, anti-IL17F, anti-TNFα antibody treatment and TNFα-deficient mice in early phase *M. tuberculosis* model. (B) Comparison of anti-IL-17A antibody treatment and TNFα-deficient mice in *M. tuberculosis* reactivation model. (C) Comparison of IL-17RA-, IL-22-, IL-17RA-IL-22- and TNFα-deficient mice in early and late phase *M. tuberculosis* model.

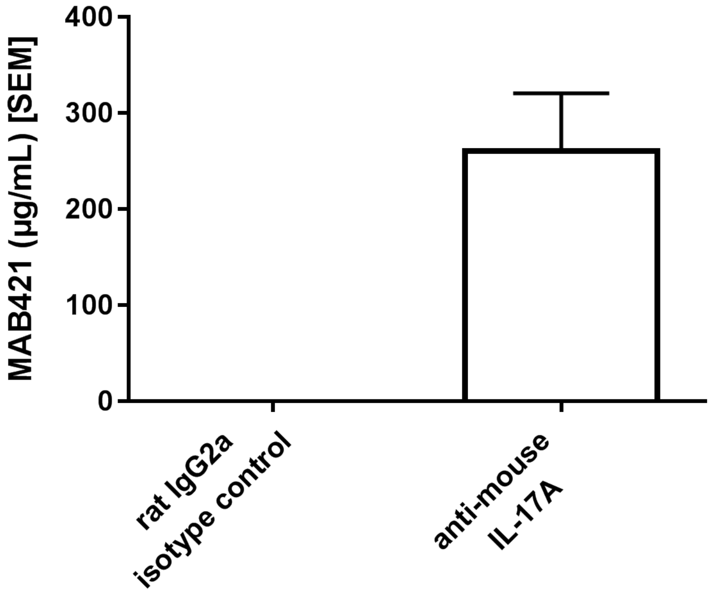

Supplementary Fig. S2. Plasma levels of anti-IL-17A antibody levels in anti-IL-17A antibody- and isotype control-treated mice at day 28 of treatment (n=10). *M. tuberculosis* infected C57BL/6 mice (H37Rv, 1000 CFU i.n.) were treated once per week with anti-mouse IL-17A antibody 500 µg per mouse (20 mg/kg i.p.), and isotype control antibody, starting 1 day before the infection.

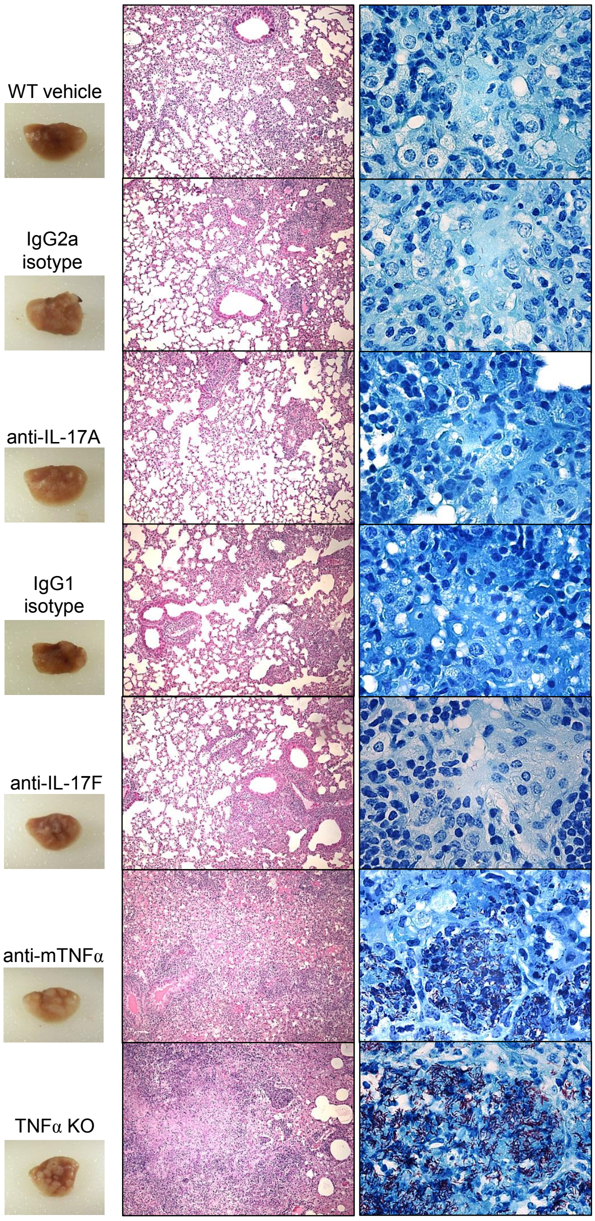

Supplementary Fig. S3. Neutralizing IL-17A or IL-17F does not compromise host response to early phase of *M. tuberculosis* infection, in contrast to TNFα neutralization. *M. tuberculosis* infected C57BL/6 mice (H37Rv, 1000 CFU i.n.) were treated once per week with anti-mouse IL-17A or IL-17F antibodies (20 mg/kg i.p.), anti-mouse TNFα antibody (10 mg/kg), respective isotype control antibodies, or vehicle, starting 1 day before the infection, and monitored for body weight. Lung pathology was assessed on day 28. Lungs of TNFα-deficient mice with large, confluent nodules are included for comparison. Microscopic examination showing extensive inflammation and necrosis in lungs of infected TNFα-deficient mice or anti-TNFα antibody treated mice (Left: haematoxylin and eosin, magnification x 50) as well as numerous acid-fast bacilli (Right: Ziehl-Nielson, magnification x 1000).

A **Chemokine and cytokine responses**

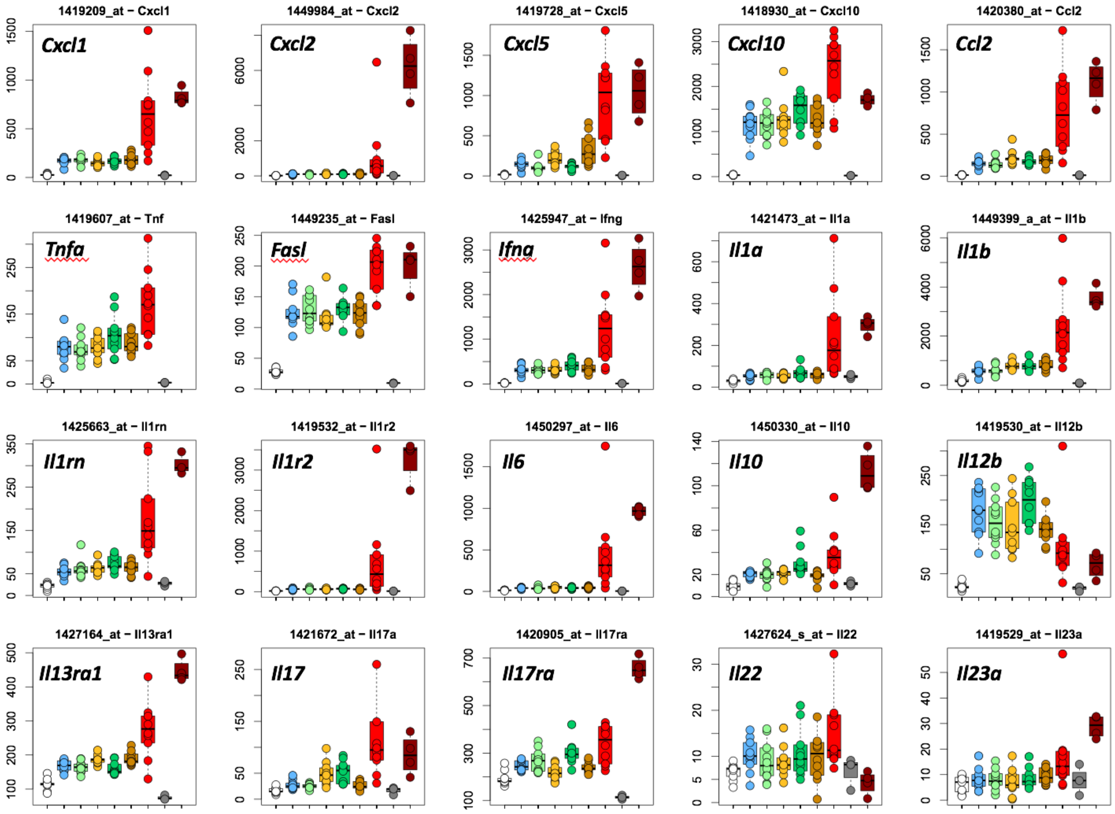

B **Host-pathogen interactions, antimycobacterial activity, and modulatory responses**

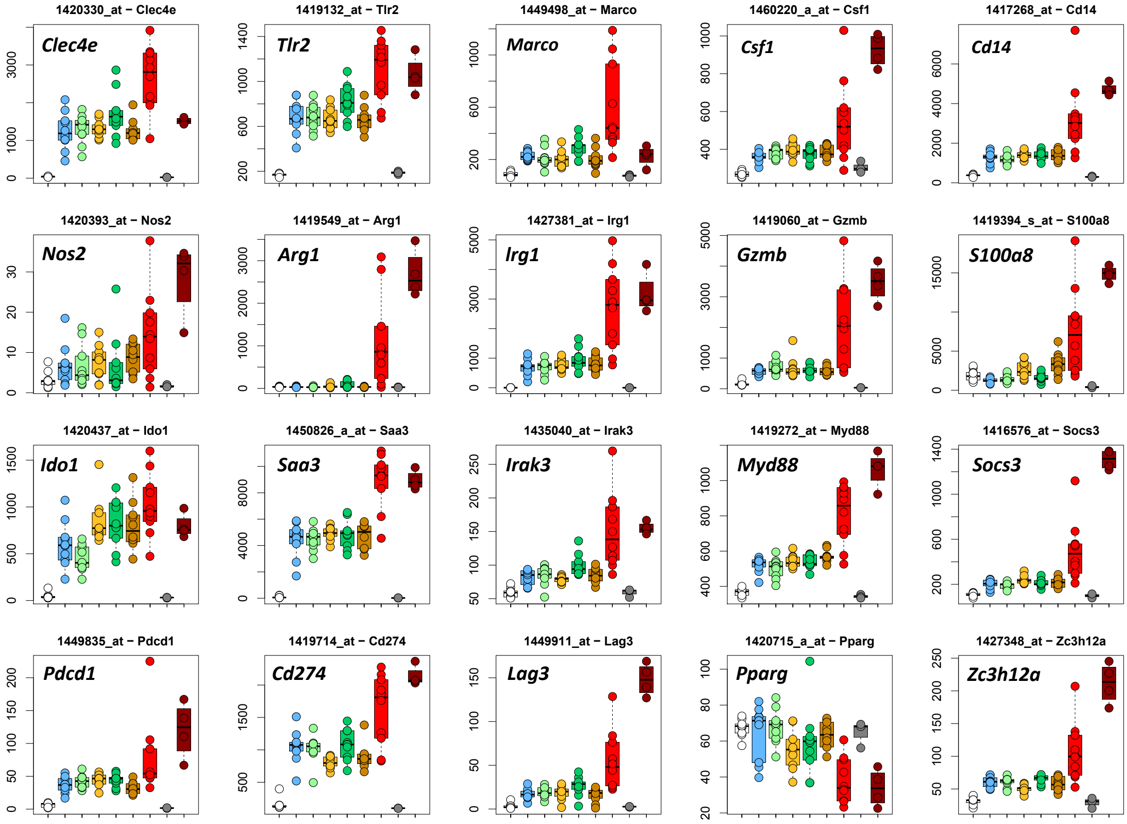

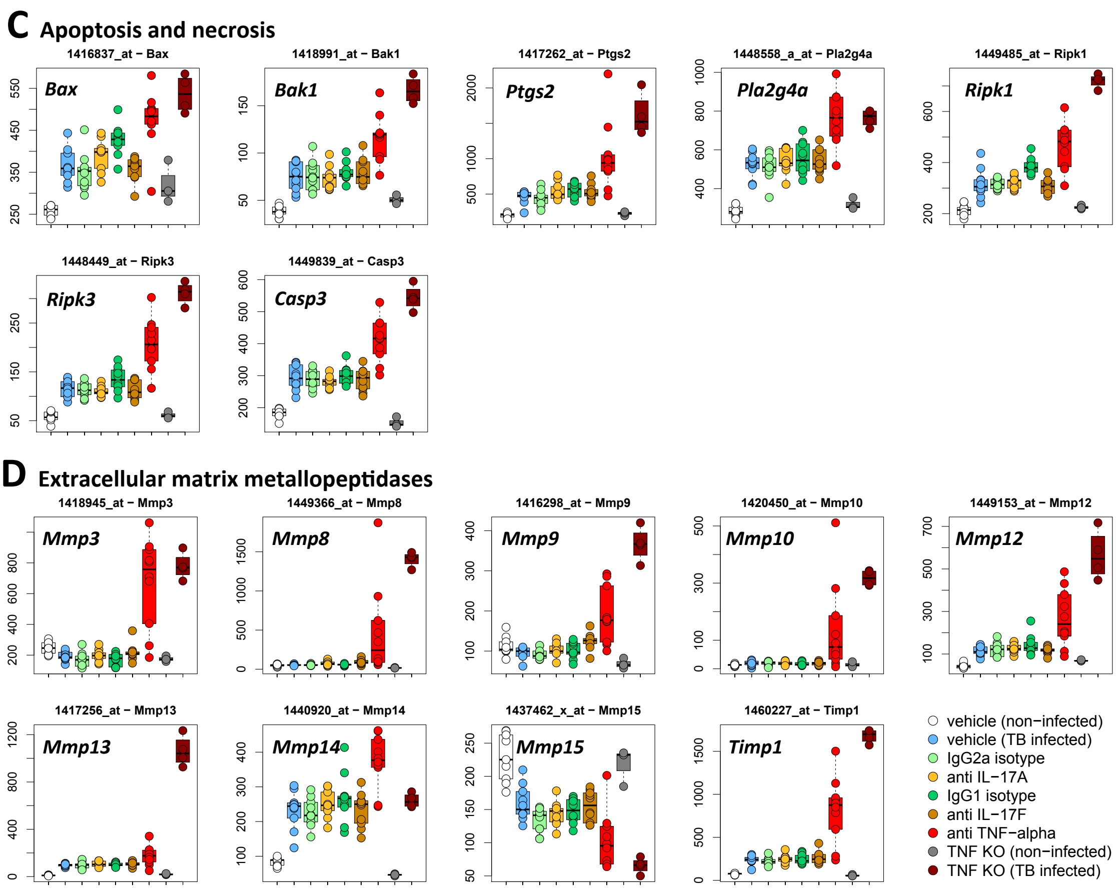

Supplementary Fig. S4. TNFα neutralization exacerbates gene expression of inflammatory markers in early phase of *M. tuberculosis* infection, in contrast to anti-IL-17A or anti-IL-17F treatment. Transcriptional profiling of lungs from *M. tuberculosis* infected C57BL/6 mice (H37Rv, 1000 CFU i.n.) treated once per week with anti-mouse IL-17A or IL-17F antibodies (20 mg/kg i.p.), anti-mouse TNFα antibody (10 mg/kg i.p.), respective isotype control antibodies, or vehicle, starting 1 day before the infection, and from non-infected C57BL/6 mice treated with vehicle or from *M. tuberculosis* infected and non-infected TNFα-deficient mice was performed on day 28 to assess molecular changes in the different conditions. Boxplots for selected genes showing the MAS5 normalized expression level for each individual animal, the lower and upper quartiles and the median for the group.

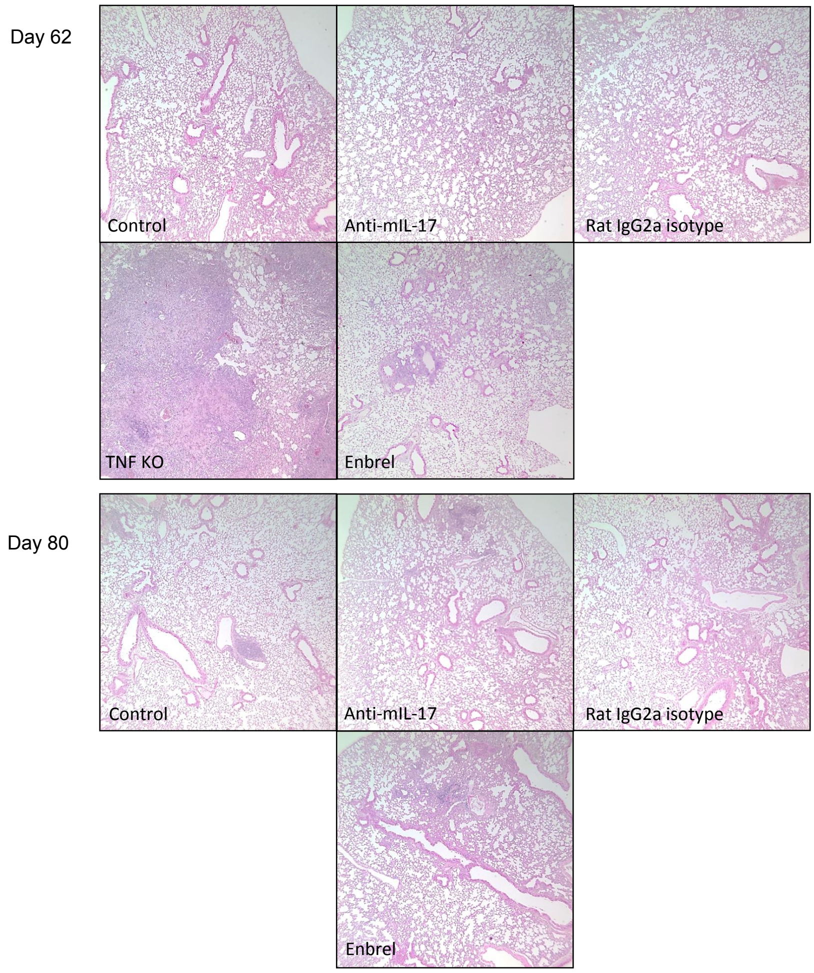

Supplementary Fig. S5. Neutralizing IL-17A does not compromise host response to *M. tuberculosis* infection in a murine reactivation model. Two weeks following *M. tuberculosis* infection of C57BL/6 mice or TNFα-deficient mice (H37Rv, 1000 CFU i.n.), mice were subsequently treated for 3 weeks with isoniazid (INH) and rifampicin (RIF) to control the infection. At 5 weeks, mice were injected i.p. weekly with an IL-17A neutralizing antibody (IgG2a; MAB421 R&D), or the respective isotype control antibody (MAB006 R&D; all 1 mg/mouse) or with Enbrel (30 mg/kg) for 4 weeks. Lung pathology was assessed on day 62 and 80. Microscopic examination showing extensive inflammation and necrosis in infected TNFα-deficient lungs with large confluent nodules on day 72 are included for comparison (haematoxylin and eosin, magnification x 50).

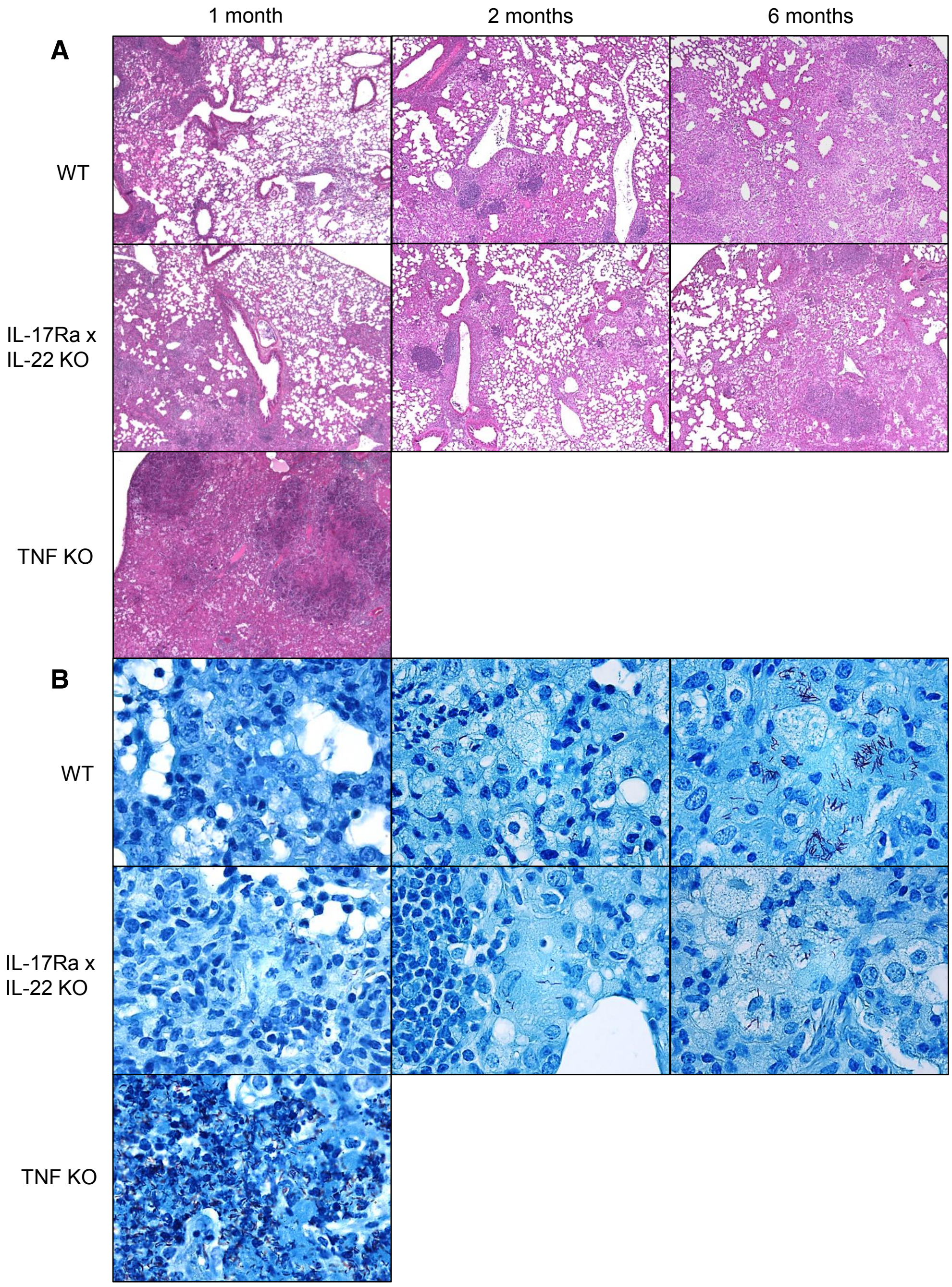

Supplementary Fig. S6. Contained chronic *M. tuberculosis* infection in the absence of IL-17RA and/or IL-22 pathways. Mice deficient for IL-17RA, IL-22, both IL-17RA and IL-22, and TNFα mice as well as wild-type mice were infected with *M. tuberculosis* (H37Rv, 1000 CFU/mouse i.n.). Lung pathology was assessed at 1, 2, and 6 months. Macroscopically large, confluent nodules were visible in all groups. Microscopic examination showed extensive inflammation with limited free alveolar space (A, haematoxylin and eosin, magnification x 50), with the presence of acid-fast bacilli (B, Ziehl-Nielson, magnification x 1000).

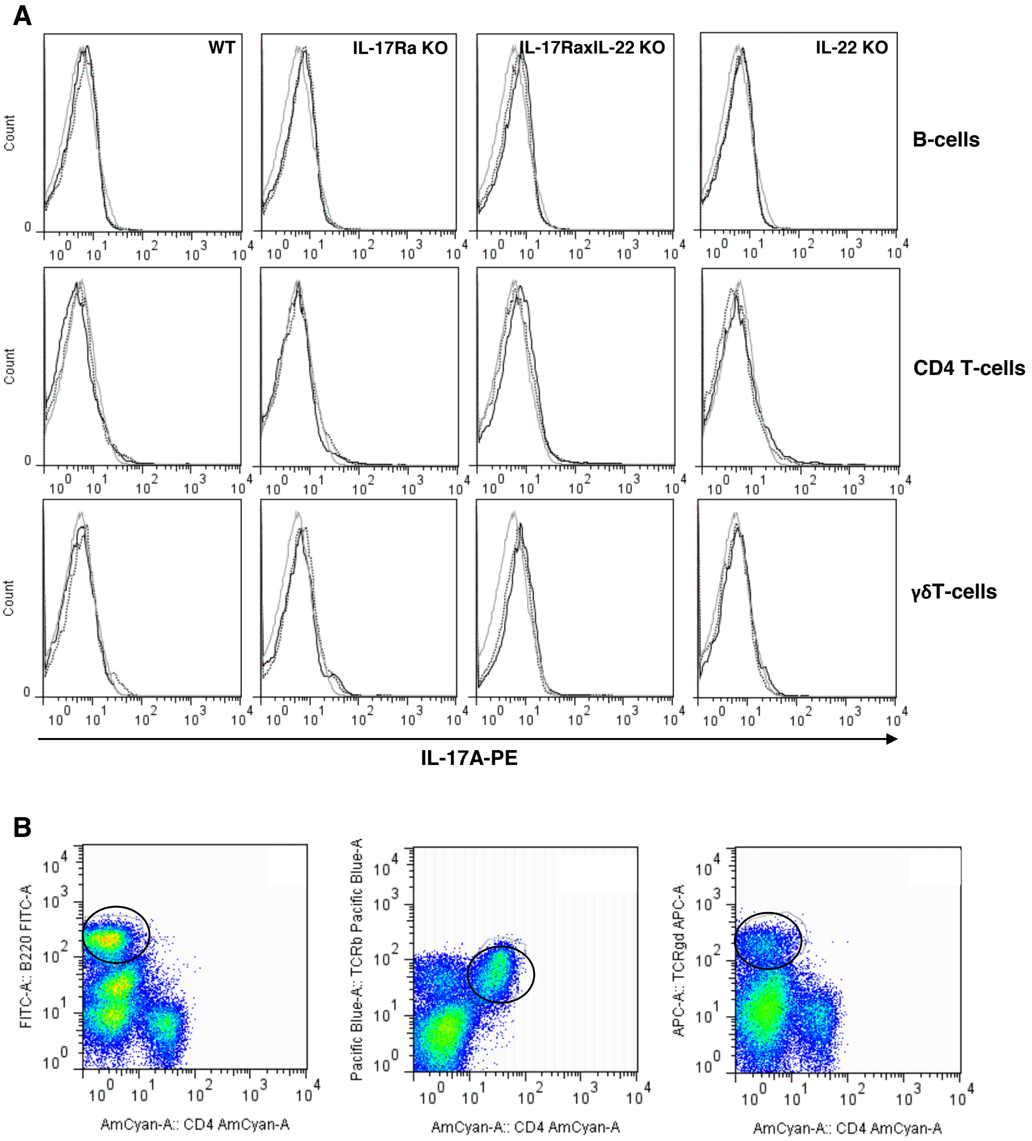

Supplementary Fig. S7. IL-17A expression by CD4⁺ T-cells and γδT cells in the absence of IL-17RA and IL-22. Lung infiltrating cells from mice deficient for IL-17RA and/or IL-22 and wild-type mice exposed to *M. tuberculosis* (1000 CFU/mouse i.n.) were analysed by flow cytometry at 2 months post-infection. Intracellular staining for IL-17A is shown for $B220^+CD4^-$ B cells, $CD4^+TCR\beta^+$ T-cells and $TCR\gamma\delta^+CD4^-$ γδT-cells, as indicated (A). Representative histograms are shown for 2 individual mice per group with isotype controls in light grey. (B) Representative dot plots are shown to illustrate the gating strategy.

Supplementary Table 1 CD4$^+$ T cells transcriptomic signature

ProbeSet	GeneSymbol	GeneName
1423135_at	Thy1	thymus cell antigen 1, theta
1452405_x_at	Trav9d-3	T cell receptor alpha variable 9D-3
1425570_at	Slamf1	signaling lymphocytic activation molecule family member 1
1421930_at	Icos	inducible T cell co-stimulator
1426168_a_at	Trav9d-3	T cell receptor alpha variable 9D-3
1426159_x_at	Tcrb-J	T cell receptor beta, joining region
1440178_x_at	Zap70	zeta-chain (TCR) associated protein kinase
1426396_at	Cd247	CD247 antigen
1448576_at	Il7r	interleukin 7 receptor
1448575_at	Il7r	interleukin 7 receptor
1421931_at	Icos	inducible T cell co-stimulator
1426772_x_at	Tcrb-J	T cell receptor beta, joining region
1420819_at	Sla	src-like adaptor
1445068_at	Malt1	mucosa associated lymphoid tissue lymphoma translocation gene 1
1448167_at	Ifngr1	interferon gamma receptor 1
1422231_a_at	Tnfrsf25	tumor necrosis factor receptor superfamily, member 25
1426044_a_at	Prkcq	protein kinase C, theta
1423760_at	Cd44	CD44 antigen
1448759_at	Il2rb	interleukin 2 receptor, beta chain
1452539_a_at	Cd247	CD247 antigen
1420659_at	Slamf6	SLAM family member 6
1422105_at	Cd3e	CD3 antigen, epsilon polypeptide
1419714_at	Cd274	CD274 antigen
1456126_at	Malt1	mucosa associated lymphoid tissue lymphoma translocation gene 1
1425226_x_at	LOC665506	T-cell receptor beta-2 chain C region-like
1418971_x_at	Bcl10	B cell leukemia/lymphoma 10
1437025_at	Cd28	CD28 antigen
1456429_at	Malt1	mucosa associated lymphoid tissue lymphoma translocation gene 1
1460651_at	Lat	linker for activation of T cells
1418770_at	Cd2	CD2 antigen
1425854_x_at	LOC665506	T-cell receptor beta-2 chain C region-like
1427532_at	Trat1	T cell receptor associated transmembrane adaptor 1
1452205_x_at	LOC665506 : Tcrb-J : Trbv1	T-cell receptor beta-2 chain C region-like : T cell receptor beta, joining region : T cell receptor beta, variable 1
1439205_at	Nfatc2	nuclear factor of activated T cells, cytoplasmic, calcineurin dependent 2
1427184_at	Tcrb-J	T cell receptor beta, joining region
1436312_at	Ikzf1	IKAROS family zinc finger 1

ProbeSet	GeneSymbol	GeneName
1451910_a_at	Cd6	CD6 antigen
1417597_at	Cd28	CD28 antigen

Supplementary Table 2 CD8$^+$ T cells transcriptomic signature

ProbeSet	GeneSymbol	GeneName
1435560_at	Itgal	integrin alpha L
1425832_a_at	Cxcr6	chemokine (C-X-C motif) receptor 6
1457917_at	Lck	lymphocyte protein tyrosine kinase
1416296_at	Il2rg	interleukin 2 receptor, gamma chain
1445399_at	Klrb1b	killer cell lectin-like receptor subfamily B member 1B
1435227_at	Bcl11b	B cell leukemia/lymphoma 11B
1417869_s_at	Ctsz	cathepsin Z
1456836_at	Itk	IL2 inducible T cell kinase
1426169_a_at	Lat2	linker for activation of T cells family, member 2
1417868_a_at	Ctsz	cathepsin Z
1417870_x_at	Ctsz	cathepsin Z
1426170_a_at	Cd8b1	CD8 antigen, beta chain 1
1451673_at	Cd8a	CD8 antigen, alpha chain
1425084_at	Gimap7	GTPase, IMAP family member 7
1416295_a_at	Il2rg	interleukin 2 receptor, gamma chain
1420421_s_at	Klrb1b	killer cell lectin-like receptor subfamily B member 1B
1440811_x_at	Cd8a	CD8 antigen, alpha chain
1428735_at	Cd69	CD69 antigen
1444078_at	Cd8a	CD8 antigen, alpha chain
1446476_at	Trav12n-2	T cell receptor alpha variable 12N-2
1460245_at	Klrd1	killer cell lectin-like receptor, subfamily D, member 1
1422632_at	Ctsw	cathepsin W
1444283_at	Gimap7	GTPase, IMAP family member 7
1437542_at	Ikzf2	IKAROS family zinc finger 2
1437939_s_at	Ctsc	cathepsin C
1419226_at	Cd96	CD96 antigen
1420404_at	Cd86	CD86 antigen
1417171_at	Itk	IL2 inducible T cell kinase
1424375_s_at	Gimap4	GTPase, IMAP family member 4
1418353_at	Cd5	CD5 antigen
1416382_at	Ctsc	cathepsin C
1422067_at	Klrb1b	killer cell lectin-like receptor subfamily B member 1B
1425335_at	Cd8a	CD8 antigen, alpha chain
1422812_at	Cxcr6	chemokine (C-X-C motif) receptor 6
1448160_at	Lcp1	lymphocyte cytosolic protein 1
1427667_s_at	Tcrb-J	T cell receptor beta, joining region
1452966_at	Bcl11b	B cell leukemia/lymphoma 11B

Supplementary Table 3 γδT cells transcriptomic signature

ProbeSet	GeneSymbol	GeneName
1450521_a_at	Tcrg-V4	T cell receptor gamma, variable 4
1422189_x_at	Tcrg-V4	T cell receptor gamma, variable 4
1455527_at	Cd163l1	CD163 molecule-like 1
1427656_at	Tcrb-J	T cell receptor beta, joining region
1449835_at	Pdcd1	programmed cell death 1
1423467_at	Ms4a4b	membrane-spanning 4-domains, subfamily A, member 4B
1420691_at	Il2ra	interleukin 2 receptor, alpha chain
1440808_x_at	Cd163l1	CD163 molecule-like 1
1422701_at	Zap70	zeta-chain (TCR) associated protein kinase
1421672_at	Il17a	interleukin 17A
1422188_s_at	Tcrg-C1 : Tcrg-C2	T cell receptor gamma, constant 1 : T-cell receptor gamma, constant 2
1425396_a_at	Lck	lymphocyte protein tyrosine kinase
1419083_at	Tnfsf11	tumor necrosis factor (ligand) superfamily, member 11
1419178_at	Cd3g	CD3 antigen, gamma polypeptide
1417620_at	Rac2	RAS-related C3 botulinum substrate 2
1422828_at	Cd3d	CD3 antigen, delta polypeptide

Supplementary Table 4 NK cells transcriptomic signature

ProbeSet	GeneSymbol	GeneName
1419026_at	Daxx	Fas death domain-associated protein
1418803_a_at	Fasl	Fas ligand (TNF superfamily, member 6)
1452352_at	Ctla2b	cytotoxic T lymphocyte-associated protein 2 beta
1417898_a_at	Gzma	granzyme A
1449235_at	Fasl	Fas ligand (TNF superfamily, member 6)
1450753_at	Nkg7	natural killer cell group 7 sequence
1449991_at	Cd244	CD244 natural killer cell receptor 2B4
1425888_at	Klra17	killer cell lectin-like receptor, subfamily A, member 17
1450495_a_at	Klrk1	killer cell lectin-like receptor subfamily K, member 1
1421304_at	Klra2	killer cell lectin-like receptor, subfamily A, member 2
1419060_at	Gzmb	granzyme B
1422280_at	Gzmk	granzyme K
1419334_at	Ctla4	cytotoxic T-lymphocyte-associated protein 4
1425005_at	Klrc1	killer cell lectin-like receptor subfamily C, member 1
1449131_s_at	Cd1d1	CD1d1 antigen
1420788_at	Klrg1	killer cell lectin-like receptor subfamily G, member 1

Supplementary Table 5 Macrophage transcriptomic signature

ProbeSet	GeneSymbol	GeneName
1449580_s_at	H2-DMb1 : H2-	histocompatibility 2, class II, locus Mb1 :

ProbeSet	GeneSymbol	GeneName
	DMb2	histocompatibility 2, class II, locus Mb2
1415983_at	Lcp1	lymphocyte cytosolic protein 1
1416952_at	Atp6v1d	ATPase, H+ transporting, lysosomal V1 subunit D
1417009_at	C1ra : C1rb	complement component 1, r subcomponent A : complement component 1, r subcomponent B
1425890_at	Ly6i	lymphocyte antigen 6 complex, locus I
1425545_x_at	H2-D1 : H2-L	histocompatibility 2, D region locus 1 : histocompatibility 2, D region locus L
1459872_x_at	H2-DMa	histocompatibility 2, class II, locus DMa
1427746_x_at	H2-K1 : LOC101056305	histocompatibility 2, K1, K region : h-2 class I histocompatibility antigen, K-D alpha chain-like
1450648_s_at	H2-Ab1	histocompatibility 2, class II antigen A, beta 1
1419128_at	Itgax	integrin alpha X
1449473_s_at	Cd40	CD40 antigen
1422962_a_at	Psmb8	proteasome (prosome, macropain) subunit, beta type 8 (large multifunctional peptidase 7)
1451721_a_at	H2-Ab1	histocompatibility 2, class II antigen A, beta 1
1435476_a_at	Fcgr2b	Fc receptor, IgG, low affinity IIb
1448591_at	Ctss	cathepsin S
1417381_at	C1qa	complement component 1, q subcomponent, alpha polypeptide
1422191_at	Cd200r1	CD200 receptor 1
1419684_at	Ccl8 : LOC100503254	chemokine (C-C motif) ligand 8 : c-C motif chemokine 8-like
1419873_s_at	Csf1r	colony stimulating factor 1 receptor
1416016_at	Tap1	transporter 1, ATP-binding cassette, sub-family B (MDR/TAP)
1453304_s_at	Ly6e	lymphocyte antigen 6 complex, locus E
1456772_at	Ncf1	neutrophil cytosolic factor 1
1448123_s_at	Tgfbi	transforming growth factor, beta induced
1448898_at	Ccl9	chemokine (C-C motif) ligand 9
1420699_at	Clec7a	C-type lectin domain family 7, member a
1438858_x_at	H2-Aa	histocompatibility 2, class II antigen A, alpha
1425519_a_at	Cd74	CD74 antigen (invariant polypeptide of major histocompatibility complex, class II antigen-associated)
1454268_a_at	Cyba	cytochrome b-245, alpha polypeptide
1450792_at	Tyrobp	TYRO protein tyrosine kinase binding protein
1437726_x_at	C1qb	complement component 1, q subcomponent, beta polypeptide
1435290_x_at	H2-Aa	histocompatibility 2, class II antigen A, alpha
1443783_x_at	H2-Aa	histocompatibility 2, class II antigen A, alpha
1422527_at	H2-DMa	histocompatibility 2, class II, locus DMa

ProbeSet	GeneSymbol	GeneName
1448632_at	Psmb10	proteasome (prosome, macropain) subunit, beta type 10
1436559_a_at	Psmd10	proteasome (prosome, macropain) 26S subunit, non-ATPase, 10
1417490_at	Ctsb	cathepsin B
1421211_a_at	Ciita	class II transactivator
1425336_x_at	H2-K1	histocompatibility 2, K1, K region
1418536_at	H2-Q7 : H2-Q9	histocompatibility 2, Q region locus 7 : histocompatibility 2, Q region locus 9
1418204_s_at	Aif1	allograft inflammatory factor 1
1444040_at	Lair1	leukocyte-associated Ig-like receptor 1
1455332_x_at	Fcgr2b	Fc receptor, IgG, low affinity IIb
1448732_at	Ctsb	cathepsin B
1450696_at	Psmb9	proteasome (prosome, macropain) subunit, beta type 9 (large multifunctional peptidase 2)
1415871_at	Tgfbi	transforming growth factor, beta induced
1450678_at	Itgb2	integrin beta 2
1421065_at	Jak2	Janus kinase 2
1421326_at	Csf2rb	colony stimulating factor 2 receptor, beta, low-affinity (granulocyte-macrophage)
1418021_at	C4b : LOC675521	complement component 4B (Chido blood group) : complement C4-B-like
1418826_at	Ms4a6b	membrane-spanning 4-domains, subfamily A, member 6B
1451462_a_at	Ifnar2	interferon (alpha and beta) receptor 2
1449858_at	Cd86	CD86 antigen
1451784_x_at	H2-D1	histocompatibility 2, D region locus 1
1418638_at	H2-DMb1 : H2-DMb2	histocompatibility 2, class II, locus Mb1 : histocompatibility 2, class II, locus Mb2
1448128_at	Ctsa	cathepsin A
1419482_at	C3ar1	complement component 3a receptor 1
1421813_a_at	Psap	prosaposin
1417025_at	H2-Eb1	histocompatibility 2, class II antigen E beta
1419132_at	Tlr2	toll-like receptor 2
1442082_at	C3ar1	complement component 3a receptor 1
1417936_at	Ccl9	chemokine (C-C motif) ligand 9
1453913_a_at	Tap2	transporter 2, ATP-binding cassette, sub-family B (MDR/TAP)
1460339_at	Psma4	proteasome (prosome, macropain) subunit, alpha type 4
1448883_at	Lgmn	legumain
1450234_at	Ms4a6c	membrane-spanning 4-domains, subfamily A, member 6C
1418465_at	Ncf4	neutrophil cytosolic factor 4
1417063_at	C1qb	complement component 1, q subcomponent, beta polypeptide

ProbeSet	GeneSymbol	GeneName
1451931_x_at	H2-K1	histocompatibility 2, K1, K region
1450378_at	Tapbp	TAP binding protein
1449360_at	Csf2rb2	colony stimulating factor 2 receptor, beta 2, low-affinity (granulocyte-macrophage)
1438629_x_at	Grn	granulin
1417721_s_at	Laptm5	lysosomal-associated protein transmembrane 5
1417189_at	Psme2	proteasome (prosome, macropain) 28 subunit, beta
1435313_at	Cd200r4	CD200 receptor 4
1451941_a_at	Fcgr2b	Fc receptor, IgG, low affinity IIb
1424041_s_at	C1s	complement component 1, s subcomponent
1456250_x_at	Tgfbi	transforming growth factor, beta induced
1436905_x_at	Laptm5	lysosomal-associated protein transmembrane 5
1425477_x_at	H2-Ab1	histocompatibility 2, class II antigen A, beta 1
1435477_s_at	Fcgr2b	Fc receptor, IgG, low affinity IIb
1449401_at	C1qc	complement component 1, q subcomponent, C chain
1427076_at	Mpeg1	macrophage expressed gene 1
1417314_at	C2 : Cfb	complement component 2 (within H-2S) : complement factor B
1421210_at	Ciita	class II transactivator
1448148_at	Grn	granulin
1424948_x_at	H2-D1 : H2-K1 : LOC101056305	histocompatibility 2, D region locus 1 : histocompatibility 2, K1, K region : h-2 class I histocompatibility antigen, K-D alpha chain-like
1449164_at	Cd68	CD68 antigen
1452431_s_at	H2-Aa	histocompatibility 2, class II antigen A, alpha
1456567_x_at	Grn	granulin
1455660_at	Csf2rb	colony stimulating factor 2 receptor, beta, low-affinity (granulocyte-macrophage)
1451544_at	Tapbpl	TAP binding protein-like
1421812_at	Tapbp	TAP binding protein
1417056_at	Psme1	proteasome (prosome, macropain) 28 subunit, alpha
1460218_at	Cd52	CD52 antigen
1423954_at	C3 : LOC100048759	complement component 3 : complement C3-like
1448731_at	Il10ra	interleukin 10 receptor, alpha
1415687_a_at	Psap	prosaposin
1418340_at	Fcer1g	Fc receptor, IgE, high affinity I, gamma polypeptide
1451683_x_at	H2-D1	histocompatibility 2, D region locus 1

Supplementary Table 6 Monocyte transcriptomic signature

ProbeSet	GeneSymbol	GeneName
1424067_at	Icam1	intercellular adhesion molecule 1
1427164_at	Il13ra1	interleukin 13 receptor, alpha 1
1416303_at	Litaf	LPS-induced TN factor
1450297_at	Il6	interleukin 6
1427736_a_at	Ccrl2	chemokine (C-C motif) receptor-like 2
1456212_x_at	Socs3	suppressor of cytokine signaling 3
1419549_at	Arg1	arginase, liver
1449731_s_at	Nfkbia	nuclear factor of kappa light polypeptide gene enhancer in B cells inhibitor, alpha
1451775_s_at	Il13ra1	interleukin 13 receptor, alpha 1
1427844_a_at	Cebpb	CCAAT/enhancer binding protein (C/EBP), beta
1419610_at	Ccr1	chemokine (C-C motif) receptor 1
1435040_at	Irak3	interleukin-1 receptor-associated kinase 3
1425434_a_at	Msr1	macrophage scavenger receptor 1
1419209_at	Cxcl1	chemokine (C-X-C motif) ligand 1
1438157_s_at	Nfkbia	nuclear factor of kappa light polypeptide gene enhancer in B cells inhibitor, alpha
1421034_a_at	Il4ra	interleukin 4 receptor, alpha
1418901_at	Cebpb	CCAAT/enhancer binding protein (C/EBP), beta
1417268_at	Cd14	CD14 antigen
1450446_a_at	Socs1	suppressor of cytokine signaling 1
1422062_at	Msr1	macrophage scavenger receptor 1
1416762_at	S100a10	S100 calcium binding protein A10 (calpactin)
1456642_x_at	S100a10	S100 calcium binding protein A10 (calpactin)
1448061_at	Msr1	macrophage scavenger receptor 1
1424302_at	Lilrb3	leukocyte immunoglobulin-like receptor, subfamily B (with TM and ITIM domains), member 3
1417876_at	Fcgr1	Fc receptor, IgG, high affinity I
1420394_s_at	Gp49a : Lilrb4	glycoprotein 49 A : leukocyte immunoglobulin-like receptor, subfamily B, member 4
1448929_at	F13a1	coagulation factor XIII, A1 subunit
1421571_a_at	Ly6c1 : Ly6c2	lymphocyte antigen 6 complex, locus C1 : lymphocyte antigen 6 complex, locus C2
1419598_at	Ms4a6d	membrane-spanning 4-domains, subfamily A, member 6D
1421228_at	Ccl7	chemokine (C-C motif) ligand 7
1455899_x_at	Socs3	suppressor of cytokine signaling 3
1420380_at	Ccl2	chemokine (C-C motif) ligand 2
1419599_s_at	Ms4a6d	membrane-spanning 4-domains, subfamily A, member 6D
1449399_a_at	Il1b	interleukin 1 beta
1457644_s_at	Cxcl1	chemokine (C-X-C motif) ligand 1
1419272_at	Myd88	myeloid differentiation primary response gene 88
1425435_at	Msr1	macrophage scavenger receptor 1
1420331_at	Clec4e	C-type lectin domain family 4, member e

ProbeSet	GeneSymbol	GeneName
1448306_at	Nfkbia	nuclear factor of kappa light polypeptide gene enhancer in B cells inhibitor, alpha
1416576_at	Socs3	suppressor of cytokine signaling 3
1441855_x_at	Cxcl1	chemokine (C-X-C motif) ligand 1
1421473_at	Il1a	interleukin 1 alpha
1419728_at	Cxcl5	chemokine (C-X-C motif) ligand 5

Supplementary Table 7 Neutrophil transcriptomic signature

ProbeSet	GeneSymbol	GeneName
1419532_at	Il1r2	interleukin 1 receptor, type II
1419394_s_at	S100a8	S100 calcium binding protein A8 (calgranulin A)
1460282_at	Trem1	triggering receptor expressed on myeloid cells 1
1448291_at	Mmp9	matrix metallopeptidase 9
1418806_at	Csf3r	colony stimulating factor 3 receptor (granulocyte)
1448914_a_at	Csf1	colony stimulating factor 1 (macrophage)
1421578_at	Ccl4	chemokine (C-C motif) ligand 4
1449366_at	Mmp8	matrix metallopeptidase 8
1421375_a_at	S100a6	S100 calcium binding protein A6 (calcyclin)
1439902_at	C5ar1	complement component 5a receptor 1
1418847_at	Arg2	arginase type II
1417074_at	Ceacam10	carcinoembryonic antigen-related cell adhesion molecule 10
1451054_at	Orm1	orosomucoid 1
1419427_at	Csf3	colony stimulating factor 3 (granulocyte)
1452521_a_at	Plaur	plasminogen activator, urokinase receptor
1448561_at	Ncf2	neutrophil cytosolic factor 2
1421291_at	Il18rap	interleukin 18 receptor accessory protein
1460220_a_at	Csf1	colony stimulating factor 1 (macrophage)
1425958_at	Il1f9	interleukin 1 family, member 9
1420407_at	Ltb4r1	leukotriene B4 receptor 1
1448756_at	S100a9	S100 calcium binding protein A9 (calgranulin B)
1450513_at	Cd33	CD33 antigen
1421734_at	Cxcr2	chemokine (C-X-C motif) receptor 2
1418674_at	Osmr	oncostatin M receptor
1418722_at	Ngp	neutrophilic granule protein
1418932_at	Nfil3	nuclear factor, interleukin 3, regulated
1452016_at	Alox5ap	arachidonate 5-lipoxygenase activating protein
1417256_at	Mmp13	matrix metallopeptidase 13
1423017_a_at	Il1rn	interleukin 1 receptor antagonist
1420904_at	Il17ra	interleukin 17 receptor A

ProbeSet	GeneSymbol	GeneName
1447284_at	Trem1	triggering receptor expressed on myeloid cells 1
1416871_at	Adam8	a disintegrin and metallopeptidase domain 8
1438148_at	Cxcl3	chemokine (C-X-C motif) ligand 3
1416298_at	Mmp9	matrix metallopeptidase 9
1450330_at	Il10	interleukin 10
1425663_at	Il1rn	interleukin 1 receptor antagonist
1418666_at	Ptx3	pentraxin related gene
1438841_s_at	Arg2	arginase type II
1449984_at	Cxcl2	chemokine (C-X-C motif) ligand 2
1427747_a_at	Lcn2	lipocalin 2
1448573_a_at	Ceacam10	carcinoembryonic antigen-related cell adhesion molecule 10
1451798_at	Il1rn	interleukin 1 receptor antagonist
1424509_at	Cd177	CD177 antigen
1420438_at	Orm2	orosomucoid 2
1448620_at	Fcgr3	Fc receptor, IgG, low affinity III
1418945_at	Mmp3	matrix metallopeptidase 3
1417795_at	Chl1	cell adhesion molecule with homology to L1CAM
1456545_at	Il18rap	interleukin 18 receptor accessory protein
1460351_at	Gm12854 : Gm5068 : S100a11	predicted gene 12854 : predicted gene 5068 : S100 calcium binding protein A11 (calgizzarin)
1422953_at	Fpr2	formyl peptide receptor 2
1438767_at	Osm	oncostatin M
1420905_at	Il17ra	interleukin 17 receptor A
1427994_at	Cd300lf	CD300 antigen like family member F
1420450_at	Mmp10	matrix metallopeptidase 10
1460227_at	Timp1	tissue inhibitor of metalloproteinase 1
1419609_at	Ccr1	chemokine (C-C motif) receptor 1

Supplementary Table 8 B cell transcriptomic signature

ProbeSet	GeneSymbol	GeneName
1435955_at	Siglecg	sialic acid binding Ig-like lectin G
1416930_at	Ly6d	lymphocyte antigen 6 complex, locus D
1417640_at	Cd79b	CD79B antigen
1456632_at	Bcl11a	B cell CLL/lymphoma 11A (zinc finger protein)
1440837_at	H2-Ob	histocompatibility 2, O region beta locus
1419907_s_at	Fcrla	Fc receptor-like A
1423226_at	Ms4a1	membrane-spanning 4-domains, subfamily A, member 1
1425062_at	Fcrl1	Fc receptor-like 1
1455530_at	Ighv14-2	immunoglobulin heavy variable 14-2
1429889_at	Faim3	Fas apoptotic inhibitory molecule 3
1456328_at	Bank1	B cell scaffold protein with ankyrin repeats 1

ProbeSet	GeneSymbol	GeneName
1419768_at	Cd22	CD22 antigen
1450912_at	Ms4a1	membrane-spanning 4-domains, subfamily A, member 1
